# Small RNA Sequencing Reveals Exosomal miRNAs Involved in the Treatment of Asthma by Scorpio and Centipede

**DOI:** 10.1155/2020/1061407

**Published:** 2020-01-14

**Authors:** Binqing Tang, Yingen Wu, Hong Fang, Yuqin Wu, Kehua Shi

**Affiliations:** ^1^Department of Respiratory Medicine, Shanghai Municipal Hospital of Traditional Chinese Medicine, Shanghai University of Traditional Chinese Medicine, Shanghai, China; ^2^Department of Internal Medicine, Longhua Hospital, Shanghai University of Traditional Chinese Medicine, Shanghai, China; ^3^Prevention and Health Care Department of TCM, Longhua Hospital, Shanghai University of Traditional Chinese Medicine, Shanghai, China

## Abstract

Asthma is a common respiratory disease with inflammation in the lungs. Exosomes and microRNAs (miRNAs) play crucial role in inflammation, whereas the role of exosomal miRNA in asthma remains unknown. Here, we aimed to identify the key exosomal miRNAs and their underlying mechanisms involved in scorpio and centipede (SC) treatment in asthma. Eighteen mice were randomly divided into three groups: control group, asthma group, and SC treatment group. Effect of SC was assessed by hematoxylin-eosin staining and real-time PCR. Exosomes from asthma and SC treatment groups were analyzed by small RNA-seq. Results revealed SC significantly alleviated the pathogenesis of asthma and suppressed the release of inflammatory cytokines. A total of 328 exosomal miRNAs were differentially expressed between the exosomes from asthma and SC-treated mice, including 118 up- and 210 downregulated in SC-treated mice. The altered exosomal miRNAs were primarily involved in the function of transcription, apoptotic process, and cell adhesion; and pathway of calcium, Wnt, and MAPK signaling. Real-time PCR verified exosomal miR-147 was downregulated, while miR-98-5p and miR-10a-5p were upregulated in SC-treated mice compared to asthma mice. Moreover, the target genes of miR-147-3p, miR-98-5p, and miR-10a-5p were mainly enriched in Wnt and MAPK inflammatory signaling. miR-10a-5p promoted the proliferation of mouse lung epithelial cells and downregulated the expression of Nfat5 and Map2k6. These data suggest SC-induced exosomal miRNAs might mediate the inflammatory signaling and might be involved in the SC treatment in asthma. The exosomal miRNAs might be promising candidates for the treatment of asthma.

## 1. Introduction

Asthma is one of the most common respiratory diseases, which affects more than 334 million people worldwide [1]. Characterized by reversible airway inflammation, airway obstruction, and airway hyperresponsiveness, asthma has the respiratory symptoms of wheeze, chest tightness, and cough [2]. The underlying mechanisms (endogenous) of asthma are complex and represents host-environment interactions that occur at different spatial scales. Genes associated with epithelial barrier dysfunction and immune responses make a major contribution to asthma [3]. Epithelial cells, dendritic cells, and idiopathic lymphocytes are involved in the pathogenesis of asthma, and infiltration of eosinophils, basophils, and mast cells was occurred in airway smooth muscle and submucosal airway [4]. In patients with chronic asthma, persistent inflammation and smooth muscle hyperplasia can lead to thickening and narrowing of the airways, which triggers coughing, shortness of breath, and even difficulty breathing [5]. The recommended medications for adults and children include inhaled glucocorticoids and long-acting beta-2 agonists, while long-acting muscarinic antagonists, leukotriene receptor antagonists, or theophylline are considered as adjunctive therapies [3]. However, there are still limitations in the treatment of asthma with these drugs. Therefore, it is urgent to explore the pathogenesis of asthma and find new medical treatments.

Recently, large numbers of insects around the world have been identified as additional sources for novel and mechanically unique therapies. Insect Chinese medicine, such as scorpion, centipede, and earth dragon, is usually used in the treatment of refractory asthma, due to their functions of dredging collaterals, activating blood circulation and removing stasis. Scorpio and centipede (SC) showed significantly improve effects on airway inflammation and remodeling in asthmatic rats [6]. A previous study revealed that insects, including SC, produce hundreds of bioactive substances in the venom, which may be clinically useful [7]. In our previous study of 78 cases of refractory asthma, we found that treatment with SC could improve clinical symptoms and lung function and reduce airway inflammation, and no adverse reactions were found. However, the underlying mechanism of SC treatment in refractory asthma is still unknown.

Exosomes are a class of extracellular vesicles with diameters of 30 to 100 nm. As a new information carrier, exosomes carries proteins, messenger RNA (mRNA) and various noncoding RNAs, such as microRNAs (miRNAs), from donor cells to recipient cells [8]. They exist in biological fluids and play pivotal roles in multiple physiological and pathological processes [9]. Recently, the crucial role of exosomes in bronchial asthma has been discovered [10]. Bronchoalveolar lavage fluid (BALF) exosomes are involved in the cytokine and leukotriene production in allergic asthma [11]. Additionally, miRNAs have also been shown to function as potential biomarkers and therapeutic target for asthma [12]. The biological roles of exosomal miRNAs have attracted significant interest in the pulmonary field [13, 14]. Exosomal miRNAs from the BALF, such as let-7 and miRNA-200, can act as novel biomarkers for asthmatic patients [15]. However, the role of exosomal miRNAs in asthma is still largely unclear.

In the present study, we aimed to investigate the exosomal miRNAs involved in the SC treatment of refractory asthma. Effect of SC on asthma mice was assessed, and the exosomal miRNAs profiling in BALF of SC treated and control asthma mouse was investigated by small RNA sequencing. The results might provide a potential exosomal miRNAs involved in alleviating the symptoms associated with asthma.

## 2. Materials and Methods

### 2.1. Animals

SPF-grade male BALB/c mice (weight 20 ± 2 g) were obtained from J Shanghai Sipul-Bikai Laboratory Animal co., Ltd (Shanghai, China). Mice were housed in under nonpathogen conditions and in an environment of 22°C with 12 h light and 12 h dark cycle. The model was established after feeding for a week. The animal experiment in this study was approved via the animal care and ethical committee of Shanghai Municipal Hospital of Traditional Chinese Medicine.

### 2.2. Asthma Establishment and SC Treatment

A total of 18 mice were randomly divided into three groups: control group, asthma group, and SC treatment group, with 6 mice in each group. Mice in asthma group and treatment group were sensitized on the 1st and 7th days, by intraperitoneal injection with 0.1 mL allergen drugs, including OVA (0.5 mg/mL) and aluminum hydroxide (2 mg/mL). The animal was motivated on day 15. Briefly, mice were placed in an airtight container filled with 2% OVA atomized fluid for 40 min once a day, for a total of 3 weeks. Mice in the control group were intraperitoneally injected with the same amount of normal saline on the 1st and 7th day. At the 15th day, they were placed in a container filled with normal saline atomizing fluid for 4 min, once a day for 3 weeks. From day 15, each group was given intragastric administration 1 h before each stimulation, once a day for 3 weeks. On days 15–36, control group and model group received 10 mL/kg normal saline, and SC treatment group received 0.625 g/kg SC solution. At the end of experiments, BALF of mice was collected and stored at −80. Mice were euthanized via CO_2_ inhalation. The left lung of mice was partially immobilized and partially frozen.

### 2.3. Hematoxylin-Eosin (H&E) Staining

Lung tissues were fixed in 10% formalin and dehydrated with different concentrations of ethanol. Tissues were cut into 4–7 *μ*m slices, placed in a 65°C constant temperature oven for 30 min, soaked in xylene I for 15 min, and then in xylene II for 15 min. The slices were soaked with 100% alcohol, 95% alcohol, 85% alcohol, and 75% alcohol for 5 min, respectively, and washed with running water for 10 min. Slices were stained with Hematoxylin aqueous solution for 5 min and eosin staining solution for 1-2 min. The slides were placed in xylene for 3 min, 2 times and sealed with neutral gum. Slides were analyzed by taking photos with a microscope (Nikon ECLIPSE Ni, Japan).

### 2.4. Exosome Extraction

Exosomes were obtained from BALF of asthma group and SC treatment group, using Exo-quick exosome precipitation kit (SBI System Biosciences, Inc.) based on the manufacturer's instruction. In brief, BLAF was centrifugated at 3000 ×*g* for 15 min under 4°C, and then the supernatant was collected, and repeat the above centrifugation. The final supernatant was collected and transferred to a new tube, then added with 252 *μ*l Exo-Quick Exosome Precipitation Solution, and incubated at 4°C for 1 h. Pelleted exosomes were obtained by centrifuging the above reaction at 1500 ×*g*, for 30 min at 4°C. The isolated exosomes were resuspended using 1 × Phosphate Buffered Saline (PBS).

### 2.5. Transmission Electron Microscopy (TEM)

The extracted exosomes were dropped on the copper network for 1 min. The floating fluid at the edge was removed by a filter paper. Exosomes were washed using ultrapure water for three times. Phosphotungstic acid of 10 *μ*l was added to the copper network and deposit for 1 min, and samples were desiccated at 20°C for 2 min. Exosomes were analyzed by a Transmission electron microscopy (NEC Electronics Corporation, JEM—1200EX).

### 2.6. Western Blot

Total protein of exosomes was isolated and the concentration was detected via a BCA assay kit (Pierce Biotechnology, Inc., Rockford, IL, USA). Equal protein from each group was loaded on 10% SDS-PAGE gel, and transferred to polyvinylidene fluoride membranes (Millipore, Bedford, MA, USA). Primary antibodies against CD63 (Santa Cruz, Santa Cruz, CA, USA; 1 : 100), and GAPDH (PB0141; 1 : 1000) was incubated with membranes at 4°C for overnight. Membranes were washed and incubated with goat antirabbit IgG-HRP secondary antibody for 2 h. Diaminobenzidine diaminobenzidine was used to stain the membranes. Enhanced chemiluminescence reagent (Thermo Fisher Scientific, Waltham, MA) was used to show the protein bands and the bands were observed by a Chemi-Doc MP system (Bio-Rad, Hercules, USA).

### 2.7. RNA Isolation, Small RNA Library Construction, and Sequencing

Three asthma mice and three SC treatment mice were involved in the RNA sequencing. Total RNA was isolated from BALF-derived exosomes via TRIzol (Invitrogen, Carlsbad, CA, USA). RNA concentration and integrity were measured by the NanoDrop 2000 spectrophotometer (NanoDrop Technologies, Inc., Wilmington, DE, USA). Illumina TruSeq RNA Sample Preparation Kit (illumina, San Diego, CA, USA) was used to establish the small RNA libraries, followed by the manufacturer's recommendations. Briefly, 1 *μ*g of total RNA was adapter-ligated with 120 nt adapter. RNA of target fragment size 135–170 nt was obtained by PAGE gel electrophoresis. Target RNA was collected with anhydrous ethanol and then reversely transcribed into cDNA and quantified with the Agilent Bioanalyzer 2100 system (Agilent Technologies, Santa Clara, CA, USA). All six libraries were sequenced on an Illumina Hiseq 2500 Genome Analyzer platform using pair-end mode, at Shanghai Yingbio biotechnology co. Ltd.

### 2.8. Data Processing

Raw reads were quality-controlled using Fast-QC (http://www.bioinformatics.babraham.ac.uk/projects/fastqc/). To identify known small noncoding RNAs, all clean reads were mapped to the miRBase database (http://www.mirbase.org/) using BWA. The reads that cannot be mapped to miRbase were then mapped to piRNA database to obtained piRNA. The transfer RNA-derived small RNAs (tsRNAs) were identified by mapping to GtRNA database (http://gtrnadb.ucsc.edu/), tRFdb (http://genome.bioch.virginia.edu/trfdb/). Differential expressed miRNAs (DEmiRNAs) were identified by the EBSeq [16], and significant differences were defined as absolute log2 fold change >1 and false discovery rate (FDR) <0.05. Potential targets of DEmiRNAs were predicted by miRanda (Score ≥150 and Energy < −20) and RNAhybrid (Energy < −25), and the final target genes were obtained by intersection of these two algorithms. Function and pathway of target genes were analyzed by Gene Ontology (GO) and the Kyoto Encyclopedia of Genes and Genomes (KEGG) database, respectively. The significance level was assessed by Fisher test and the significant enrichment was obtained by *P*-value <0.05. GO includes three classes: biological process (BP), cellular component (CC), and molecular function (MF).

### 2.9. Cell Culture

Mouse lung epithelial cells (TC-1 JHU-1) were purchased from Procell (Wuhan, China). TC-1 cells were incubated in Dulbecco-modified Eagle medium (DMEM; Life Technologies, Carlsbad, CA, USA) supplemented with 5% fetal bovine serum (FBS; Hyclone, Logan, UT, USA) and penicillin/streptomycin 100 IU/mL. Cells were cultured at 37°C, in a humidified atmosphere of 5% CO_2_ and 95% air.

### 2.10. Cell Proliferation Assays

Cell proliferation was evaluated by CCK-8 assay. Cells were harvested after 48 h of miRNA mimics or inhibitor transfection and cultured in 96-well plates (Corning, NY, USA) with 2 × 10^4^ cells/ml. CCK-8 solution (Beyotime, Shanghai, China) was added and the OD 450 was measured after culturing for 0, 24, 48, 72, or 96 h.

### 2.11. Validation of Exosomal miRNAs by Real-Time PCR

To identify key miRNAs associated with SC treatment in asthma, three exosomal miRNAs were selected for real-time PCR in six asthma mice and six SC-treated asthma mice, based on high their changed fold and abundance. RNA was isolated from BALF-derived exosomes of six asthma model mice and six SC treatment mice using TRIzol (Invitrogen, Carlsbad, CA, USA). Synthesis of cDNA was performed by specific reverse primer using High-Capacity cDNA Reverse Transcription Kit (Thermo Fisher, CA, USA). Real-time PCR was performed using SYBR Green Real-time PCR Master Mix (TOYOBO, No. QPK-201) and run on an ABI Q6 Real-time PCR System (Applied Biosystems Inc, USA). All reactions were performed in triplicate. The PCR process was as follows: one cycle of 95°C for 10 min, then 40 cycles of 95°C for 5 s, and annealing and extension at 55–58°C for 30 s. Data were analyzed using the 2^−ΔΔCt^ method. U6 was used as the reference genes, and primer sequences are shown in [Supplementary-material supplementary-material-1] of the Supplementary Materials.

### 2.12. Statistical Analysis

Statistical analysis and column diagram establishment were performed by GraphPad 7.0 (GraphPad Software, La Jolla, CA, USA). Data was presented as mean ± standard deviation (SD). Statistical comparisons between two groups were analyzed by unpaired *t*-test, and comparisons between three groups were analyzed by one-way ANOVA post Turkey test. The data were considered significant at *P*-values <0.05 (^*∗*^) or highly significant at *P* < 0.01 (^*∗∗*^).

## 3. Results

### 3.1. SC Alleviates Pathogenesis and Inflammation in Asthma Mice

**Figure 1:**
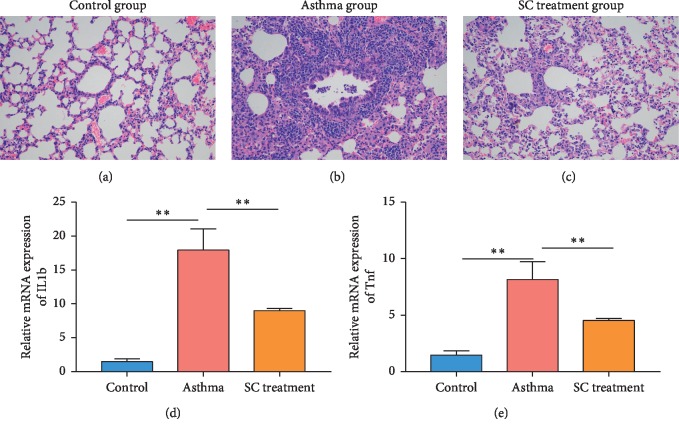
Scorpio and centipede (SC) improved pathology and inflammation of asthma in mice. (a–c) Histopathological analysis was performed by hematoxylin-eosin (H&E) staining. Enlargement factor is 200 (d and e). Expression of inflammatory factor was detected by real-time PCR. *n* = 6, two samples *t*-test, ^*∗∗*^*P* < 0.01.

The pathogenesis of sham control mice, asthmatic mice, and SC-treated mice were analyzed by H&E staining of lung tissue. Compared with the control mice, the asthma mice showed inflammatory cells (including eosinophils, neutrophils, and lymphocytes) seriously infiltrated bronchial submucosa, bronchi, and perivascular (Figures 1(a) and 1(b)). The asthma mice also displayed rupture of epithelial cell, thickening of bronchial wall and basement membrane, and irregular morphology. After SC treatment, the extent of inflammatory cell infiltration and inflammation in the lung tissues were decreased (Figure 1(c)), as compared to the asthma group. Additionally, we also detected the expression of inflammatory cytokines. The expression of IL1b and Tnf was significantly increased in the asthma group compared to that in the sham control group (Figures 1(d) and 1(e)). SC treatment significantly reduced the expression of IL1b and Tnf in lung tissues of asthma mice.

### 3.2. Characterization of the BALF-Derived Exosomes

**Figure 2:**
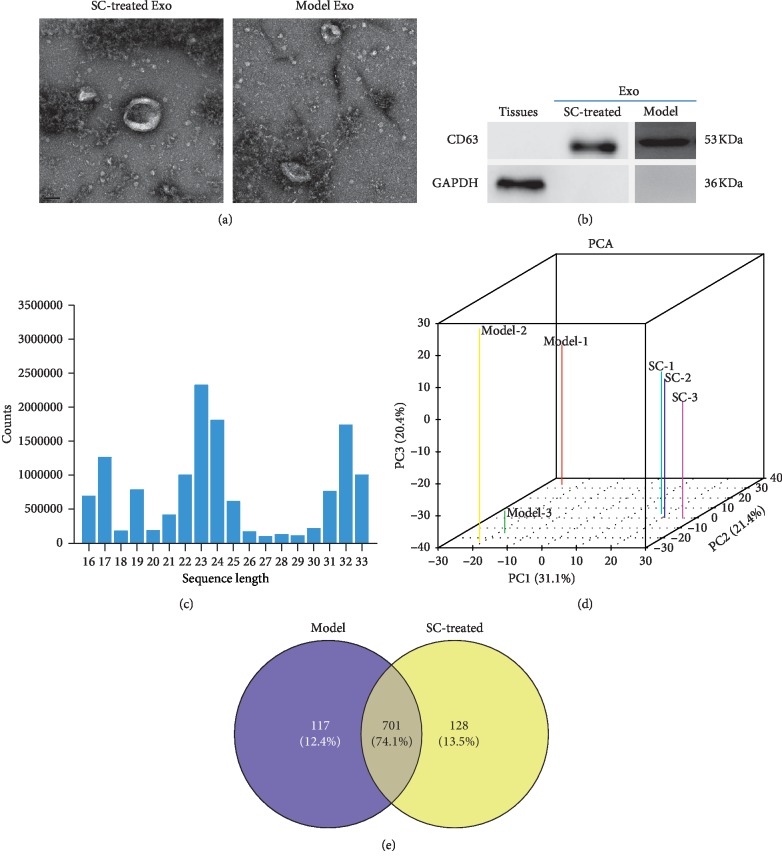
Exosomal identification and small RNA sequencing data. (a) Exosomes were analyzed by transmission electron microscopy (scale bar, 100 nm). (b) Western blot was used to measure the expression of exosomal marker in lung tissues and exosomes from bronchoalveolar lavage fluid of mice. (c) Length distribution of esosomal microRNAs (miRNAs). (d) Principal component analysis (PCA) of exosomal miRNAs from asthma group and SC treatment group. (e) Venn diagram analysis of common and unique exosomal miRNAs in the asthma group and SC treatment group. Model represents asthma group and SC represents SC treatment group.

To identify the purified exosomes, the BALF exosomes were observed by TEM which showed that exosomes were spherical particles with a complete membrane structure and with diameter of 100–150 nm (Figure 2(a)). Western blot showed that exosomes expressed the exosomal marker protein CD63 (Figure 2(b)). This result suggests the successful isolation of BALF exosomes from SC treatment and asthma model control.

### 3.3. Overview of the Small RNA Sequencing

A total of 263,659,786 raw reads were obtained from the six libraries, which generated 82,144,903 clean reads with an average of 82,144,903 clean reads per library ([Supplementary-material supplementary-material-1]). An average of 13,690,817 reads per library were mapped to the miRbase, with mapped rate from 0.374% to 0.412 for each library ([Supplementary-material supplementary-material-1]). Approximately 4407874 reads were uniquely mapped per library, with an average uniquely mapped rate of 0.32. Additionally, reads distribution showed that 2 peaks appeared at 21–23 nt and 30–33 nt, respectively, in BALF exosomes (Figure 2(c)). Principle component analysis (PCA) revealed that the principal component of miRNAs in BALF derived exosomes can significantly the discriminate asthma group and SC treatment group (Figure 2(d)). To focus on the highly represented miRNAs in exosomes, the miRNAs that have counts greater than 10 in at least one sample were considered as expressed miRNAs. As a result, 828 and 817 miRNAs were identified in the exosomes from asthma mouse and SC-treated asthma mouse, respectively (Figure 2(e)). Among the expressed miRNAs, 701 (74.1%) were shared in both groups, while 117 (12.4%) and 128 (13.5%) miRNAs were uniquely expressed in asthma and SC-treated asthma mice, respectively.

### 3.4. Differentially Expressed miRNAs in Exosomes

**Figure 3:**
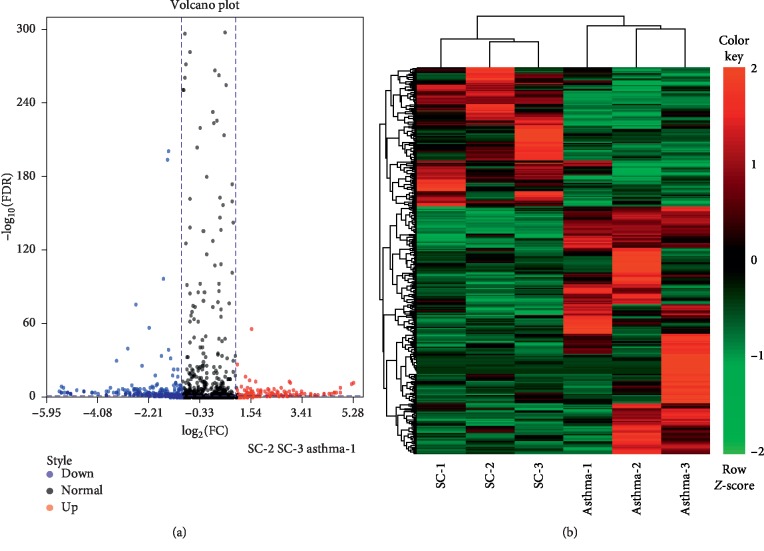
Analysis of differentially expressed miRNAs in exosomes between asthma group and SC treatment group. (a) Volcano plot analysis of differentially expressed miRNAs in bronchoalveolar lavage fluid- (BALF-) derived exosomes between the asthma group and SC treatment group. The blue point represents a downregulated exosomal miRNA and the red point represents an upregulated exosomal miRNA in the SC treatment group compared to the asthma group. (b) Heat map analysis differentially expressed miRNAs in BALF-derived exosomes between asthma group and SC treatment group. Green represents low expression level, red represents high expression level.

To identify exosomal miRNAs associated with the SC treatment in asthma, differentially expressed miRNAs (DEmiRNAs) between asthma and SC-treated asthma mice were analyzed. A total of 328 DEmiRNAs were identified between the exosomes from asthma and SC-treated asthma mice, including 118 up- and 210 downregulated miRNAs in the exosomes from SC-treated asthma mice compared to that from asthma mice (Figure 3(a)). Heat map showed the expression of DEmiRNAs was clustered into two clusters, including asthma group and SC-treated asthma group, respectively (Figure 3(b)).

### 3.5. Function and Pathway Analysis of Differentially Expressed miRNAs in Exosomes

**Figure 4:**
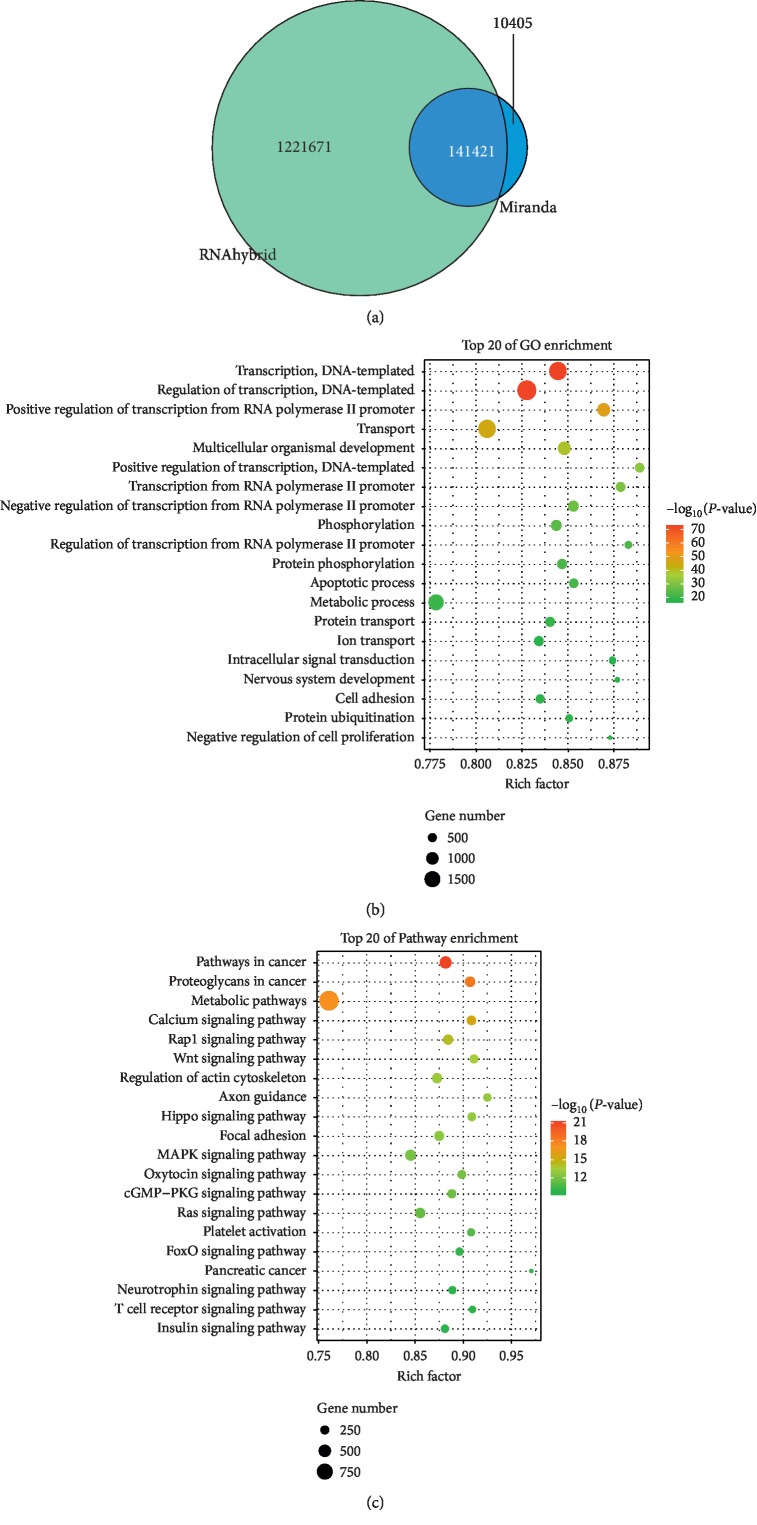
Function and pathway analysis of target genes of different exosomal miRNAs. (a) Target gene analysis of differentially expressed miRNAs. Number represents the target gene numbers of differentially expressed miRNAs using RNAhybrid and Miranda. (b) Gene Ontology (GO) analysis for target genes of differentially expressed miRNAs in BALF-derived exosomes between asthma group and SC treatment group. (c) Kyoto Encyclopedia of Genes and Genomes (KEGG) for target genes of differentially expressed miRNAs in BALF-derived exosomes between the asthma group and SC treatment group. The circle size represents the number of enriched genes and the color represents *P* value.

To uncover the functions and mechanisms of the DEmiRNAs carried by exosomes, target genes of the DEmiRNAs were predicted, and GO and KEGG enrichment was performed for target genes. A total of 141,421 genes were predictably targeted by the DEmiRNAs (Figure 4(a)). GO analysis showed that DEmiRNAs were mainly associated with the function of transcription, apoptotic process, cell adhesion, transport, and cell proliferation (Figure 4(b)). KEGG enrichment showed the targeted genes of DEmiRNAs were primarily involved in the pathway of metabolic pathways, calcium signaling pathway, Rap1 signaling pathway, Wnt signaling pathway, MAPK signaling pathway, cGMP-PKG signaling pathway, Ras signaling pathway, and FoxO signaling pathway (Figure 4(c)).

### 3.6. Validation of Differentially Expressed miRNAs from BALF Exosomes

**Figure 5:**
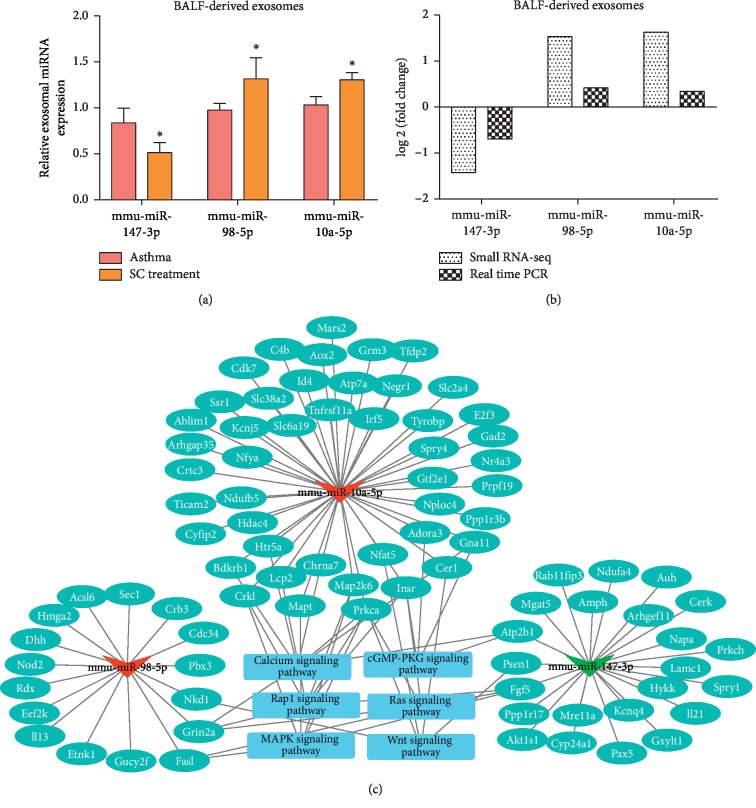
Expression and target gene analysis of the key exosomal miRNAs. (a) Candidate exosomal miRNAs were verified by real time PCR. *n* = 6, two samples *t*-test, ^*∗*^*P* < 0.05. (b) Change tendency comparison between the results of real-time PCR and small RNA-seq. Fold change: the fold change in SC treatment group compared to the asthma group. (c) Network analysis of miRNA-mRNA-pathway. Triangles, circles, and boxes represent miRNA, mRNA, and pathway, respectively. Red represents upregulated and green represents downregulated in SC treatment group compared to the asthma group.

To identify key exosomal miRNAs involved in SC treatment of asthma, three candidate miRNAs (mmu-miR-147-3p, mmu-miR-98-5p, and mmu-miR-10a-5p) were selected for real-time PCR in BALF exosomes, based on their high changed fold and abundance. In accordant with the RNA sequencing results, real-time PCR indicated that mmu-miR-147-3p was downregulated, while mmu-miR-98-5p and mmu-miR-10a-5p were upregulated in the BALF exosomes from the SC-treated mice compared to that from the asthma mice (Figures 5(a) and 5(b)).

### 3.7. Target Gene and Pathway Analysis of Differently Exosomal miRNAs

Using Miranda and RNAhybrid, we found 68, 77, and 151 genes were targeted by mmu-miR-147-3p, mmu-miR-98-5p, and mmu-miR-10a-5p, respectively ([Supplementary-material supplementary-material-1]). The target genes of the three miRNAs that were significantly enriched in the KEGG terms were used for network construction (Figure 5(c)). Importantly, the target genes *cerberus 1 (Cer1), nuclear factor of activated T cells 5 (Nfat5), NKD inhibitor of WNT signaling pathway 1 (Nkd1), presenilin 1 (Psen1),* and *protein kinase C alpha (Prkca)* were enriched in the Wnt signaling pathway; *CRK like proto-oncogene (Crkl), microtubule associated protein tau (Mapt), protein kinase C alpha (Prkca), fibroblast growth factor 5 (Fgf5)*, and *mitogen-activated protein kinase kinase 6 (Map2k6)* were enriched in MAPK signaling pathway.

### 3.8. mmu-miR-10a-5p Promotes Cell Proliferation of Mouse Lung Epithelial Cells

**Figure 6:**
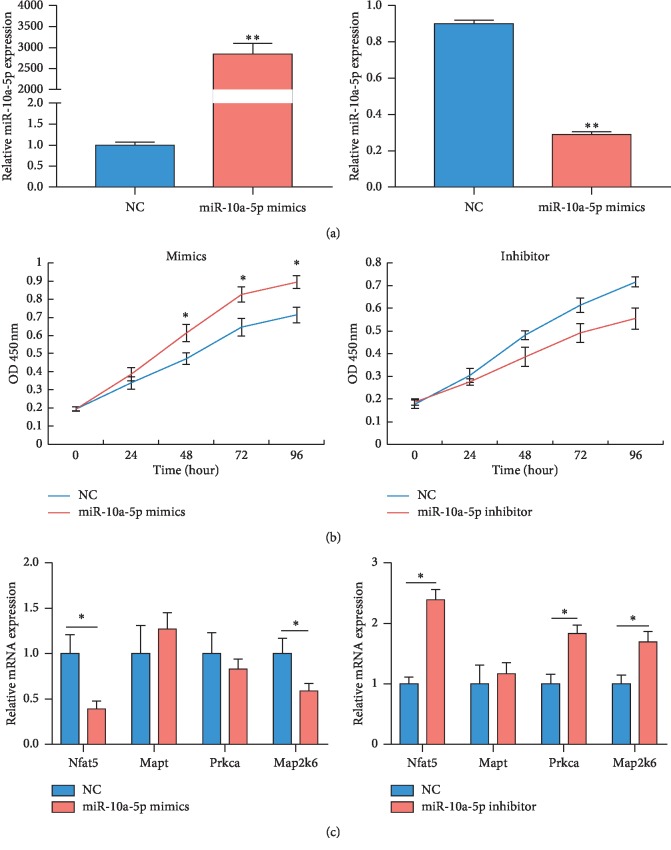
Functional and target gene validation of mmu-miR-10a-5p. (a) The transfection effect of mmu-miR-10a-5p mimics and inhibitor in mouse lung epithelial cells was verified by real-time PCR. (b) The effect of mmu-miR-10a-5p on proliferation of mouse lung epithelial cells was verified by CCK-8 assays. (c) The target gene expression of mmu-miR-10a-5p was verified by real-time PCR. *n* = 3, two samples *t*-test, ^*∗*^*P* < 0.05, ^*∗∗*^*P* < 0.01.

To investigate the function and mechanism of candidate miRNAs, mmu-miR-10a-5p was selected for further study, due to its high significant. Expression of miR-10a-5p was successfully increased in the mouse lung epithelial cells transfected with miR-10a-5p mimics, while decreased in the cells transfected with miR-10a-5p inhibitor, compared to the control (Figure 6(a)). CCK-8 assays showed miR-10a-5p mimics significantly promoted the proliferation of mouse lung epithelial cells, while miR-10a-5p inhibitor inhibited the proliferation of mouse lung epithelial cells (Figure 6(b)). Moreover, the predicted target genes, including Nfat5 and Map2k6, were significantly decreased by miR-10a-5p mimics (Figure 6(c)), while miR-10a-5p inhibitor remarkably upregulated the expression of Nfat5, Mapt, and Map2k6.

## 4. Discussion

Asthma is a common airway disease, which is characterized by chronic inflammatory and affects approximately 7.5 percent of adults [17]. SC are two antiinflammatory Chinese medicines, which are gradually used in the therapy of refractory asthma in China. Scorpion improves collagen-induced arthritis by reducing inflammatory response, via downregulating Tnf and IL-1b in rats [18]. Moreover, SC displays significantly attenuating effects on airway inflammation and remodeling in asthmatic rats [6]. Additionally, the effective components of centipede mainly include protein, peptide, carbohydrate, fatty acid, amino acid, trace, and elements. Extraction of *Scolopendra subspinipes mutilans* can inhibit inflammatory and neuropathic pain in sciatic nerve crush injury rats [19]. Centipede extraction showed the suppressed effects on inflammation and may partly via inhibition of the NF-*κ*B signaling pathway [20]. The acid protein of centipede has a significant inhibitory effect on the apoptosis of cardiomyocytes induced by angiotensin, while the polypeptide of centipede has good analgesic activity. Although the active ingredient of scorpion is unclear, a polypeptide extracted from scorpion venom suppresses angiogenesis and angiogenesis-dependent tumor growth [21]. In the present study, SC alleviated pathological characteristics of asthma mice, such as reducing the infiltration of inflammatory cells in lung tissues. Furthermore, SC decreased the expression of inflammatory factors, including Tnf and IL-1b in lung tissues of asthma mice. These evidences revealed that SC might be an effective drug to treat asthma, while more research is needed to investigate the specific active ingredients of SC in the treatment of asthma.

Exosomes and miRNAs have been shown to modulate multiple genes and signaling pathways in inflammatory responses [22, 23]. Increasing evidence demonstrates that miRNAs play a crucial role in asthma, and several asthma-related miRNAs have been identified [24], whereas the role of exosomal miRNAs in asthma, especially their functions in the asthma treatment, remains unclear. Levänen et al. showed that 24 exosomal miRNAs are differentially expressed between BLAF of asthmatics and healthy volunteers [15]. In bronchial asthma mice, epithelial cells-derived exosomes can inhibit the generation of inflammation-induced exosomes [25], indicating that exosomes may be useful for asthma treatment. In the present study, we found 328 esoxomal miRNAs altered between the asthma and SC-treated mice. Real-time PCR verified that exosomal mmu-miR-147-3p was downregulated and mmu-miR-98-5p and mmu-miR-10a-5p were upregulated after SC treatment. miR-147 has been shown to be induced by toll-like receptor and modulates inflammatory responses in mice macrophages [26]. miR-147 also reduces the production of inflammatory proteins induced by TLR2, TLR3, and TLR4-mediation [27]. miRNA-98 has been implicated to be involved in asthma by modulating peripheral B cells which is an important immune regulatory cell, via interfering the expression of thrombospondin 1 [28]. miR-10a mediates airway hyperresponsiveness by suppresses cell proliferation of airway smooth muscle, via targeting BDNF in asthmatic rats [29]. These evidences indicate exosomal miR-147, miR-98-5p, and miR-10a might be involved in the SC-treated process.

To investigate the mechanism of exosomal miRNAs in the treatment of asthma by SC, we analyzed the target genes and signaling pathway of differentially expressed miRNAs. We found the target genes of miR-147, miR-98-5p, and miR-10a were enriched in the pathway of Wnt and MAPK signaling pathway. Recent findings showed canonical and noncanonical Wnt pathways play important roles in regulation of inflammatory responses, especially in asthma [30]. Furthermore, Wnt pathways are associated with airway remodeling, cell growth of smooth muscle, and metaplasia of goblet cell [31]. Suppression of Wnt antagonist significantly improves house dust mite-induced asthma, by impairing Th2 cell cytokine secretion and leukocyte infiltration [32]. Additionally, MAPK pathway also has important effect on inflammatory responses and is involved in asthma. Inhibition of p38 MAPK might improve the corticosteroid insensitivity via modulating the release of IL-1b and IL-8 in patients with severe asthma [33]. Ginkgolide B acts as an antiInflammatory drug for asthma by inhibiting the kinase/MAPK pathway and regulating extracellular T-helper 2 cytokines in asthma mouse [34]. JAX2 improves bronchial asthma suppressing the MAPK/NF-κB inflammatory signaling pathway [35]. The present study showed that SC-induced exosomal miR-147, miR-98-5p, and miR-10a can target Wnt and MAPK pathways, suggesting exosomal miR-147, miR-98-5p, and miR-10a might mediate inflammatory pathways during the SC treatment in asthma.

In conclusion, SC can reduce the inflammatory infiltration in the lung tissues of asthma and suppress the release of inflammatory cytokines, including IL1b and Tnf. Furthermore, 328 exosomal miRNAs were differentially expressed between the asthma and SC-treated mice. SC-induced alteration of exosomal miR-147, miR-98-5p, and miR-10a-5p was verified by real-time PCR, and these miRNAs were mainly involved in the Wnt and MAPK signaling pathways. These data might provide some novel molecular targets for the treatment of asthma.

## Data Availability

The datasets used and/or analyzed during the current study are available from the corresponding author on reasonable request.

## Supplementary Materials

Supplementary Materials Table S1: statistics of the small RNA-seq data. Table S2: statistics of the mapped reads. Table S3: target gene analysis for miR-147, miR-98-5p, and miR-10a.
