# Supplementary material for: Small RNA Sequencing Reveals Exosomal miRNAs Involved in the Treatment of Asthma by Scorpio and Centipede
Source: Biomed Res Int. 2020 Jan 14;2020:1061407. doi: 10.1155/2020/1061407 (PMC6985928; doi:10.1155/2020/1061407)
Supplement: Supplementary Materials — Table S1: statistics of the small RNA-seq data. Table S2: statistics of the mapped reads. Table S3: target gene analysis for miR-147, miR-98-5p, and miR-10a. [file 1061407.f1.pdf]

Supporting information:

Table S1 Statistics of the small RNA-seq data.

SampleName	TotalReads_Before	TotalBase_Before	TotalReads_After	TotalBase_After	ReadsFilter%	BaseFilter%	GC%_Before	GC%_After	
xiaochuan1-1	21005788	3150831317	5949406	139237445	0.283226985	0.044190701	52	51	
xiaochuan1-2	49999154	7499789222	13814335	332209556	0.276291375	0.044295852	53	51	
xiaochuan1-3	52447205	7866989228	14274545	343213067	0.272169794	0.043626991	53	51	
zhiliao3-1	48740485	7310991322	16547800	397513284	0.3395083316	0.054372009	55	50	
zhiliao3-2	43724012	6558527069	14912751	355214848	0.341065477	0.054160766	53	50	
zhiliao3-3	47743142	7161391922	16646066	396053480	0.348658787	0.055303981	55	50	
	263659786	39548520080	82144903	1963441680	1.860920734	0.2959503	321	303	0
xiaochuan1-1	21005788	3150831317	5949406	139237445	0.283226985	0.044190701	52	51	
xiaochuan1-2	49999154	7499789222	13814335	332209556	0.276291375	0.044295852	53	51	
xiaochuan1-	52447205	7866989228	14274545	343213067	0.272169794	0.04362699	53	51	

3						1			
zhiliao3-1	48740485	7310991322	16547800	397513284	0.3395083316	0.054372009	55	50	
zhiliao3-2	43724012	6558527069	14912751	355214848	0.341065477	0.054160766	53	50	
zhiliao3-3	47743142	7161391922	16646066	396053480	0.348658787	0.055303981	55	50	
	43943297.67	6591420013	13690817.17	327240280	0.310153456	0.04932505	53.5	50.5	#DIV/0!

Table S2 Statistics of the mapped reads.

#Item	xiaochuan1-1.miRNA	xiaochuan1-2.miRNA	xiaochuan1-3.miRNA	zhiliao3-1.miRNA	zhiliao3-2.miRNA	zhiliao3-3.miRNA
All	5949406	13814335	14274545	16547800	14912751	16646066
UnMapped	3530944	8592926	8935702	9847559	8773099	9817506
Mapped	2418462	5221409	5338843	6700241	6139652	6828560
MappedRate	0.407	0.378	0.374	0.405	0.412	0.41
UniqueMapped	2022768	4388023	4479734	5316953	4862081	5377685
UniqueMappedRate	0.34	0.318	0.314	0.321	0.326	0.323
RepeatMapped	395694	833386	859109	1383288	1277571	1450875
AllBase	139240287	332215819	343219844	397520703	355221681	396060770
UnMappedBase	87062153	219660031	228194796	252506021	222441146	248474344
MappedBase	52178134	112555788	115025048	145014682	132780535	147586426
UniqueMappedBase	43538937	94340535	96230413	114868897	104936553	116049333
RepeatMappedBase	8639197	18215253	18794635	30145785	27843982	31537093

InsertSize	48	48	48	47	47	47

Table S3 Target gene analysis for miR-147, miR-98-5p and miR-10a.

QueryID	SubjectID	OverlapInfo	Energy_Miranda	Score_Miranda	StartSubject_Miranda	Endsubject_Miranda	Energy_RNAhybrid	P-Value_RNAhybrid	StartSubject_RNAhybrid	Endsubject_RNAhybrid	AccID	Symbol	GeneType	Location	Strand	KeggID	Description
mmu-miR-147-3p	Pax5	Miranda_RNAhybrid	-23.77	157	2248	2269	-29.1	0.528892	2248	2270	NM_008782.2	Pax5	mRNA	chr4:44531506-44710440	-	mmu:18507	Paired box protein Pax-5
mmu-miR-147-3p	Kcnq4	Miranda_RNAhybrid	-25.16	157	847	867	-30.3	0.11836	850	868	NM_001081142.1	Kcnq4	mRNA	chr4:120696134-120747491	-	mmu:60613	Potassium voltage-gated channel subfamily KQT member 4
mmu-miR-147-3p	Kpna6	Miranda_RNAhybrid	-22.47	155	88	109	-29.3	0.407137	79	110	NM_008468.4	Kpna6	mRNA	chr4:129643985-129662500	-	mmu:16650	Importin subunit alpha-7
mmu-miR-147	Eif4g3	Miranda_RNAhy	-21.84	155	476	494	-25.4	0.426623	478	495	NM_172703.3	Eif4g3	mRNA	chr4:137993013-1382064	+	mmu:230861	Eukaryotic translation initiation

-3p		brid												47			factor 4 gamma 3
mmu-miR-147-3p	Sh2d5	Miranda_RNAhybrid	-25.13	167	1481	1502	-27.3	0.380039	1483	1503	NM_001099631.1	Sh2d5	mRNA	chr4:138250411-138260968	+	mmu:230863	SH2 domain-containing protein 5
mmu-miR-147-3p	Ndufa4	Miranda_RNAhybrid	-26.74	165	40	60	-29.6	0.003384	42	60	NM_010886.2	Ndufa4	mRNA	chr6:11900373-11907446	-	mmu:17992	Cytochrome c oxidase subunit NDUFA4
mmu-miR-147-3p	Ppp1r17	Miranda_RNAhybrid	-20.27	159	1110	1131	-29.6	0.091765	1002	1032	NM_011153.3	Ppp1r17	mRNA	chr6:56017515-56032688	+	mmu:19051	Protein phosphatase 1 regulatory subunit 17
mmu-miR-147-3p	Fbxl14	Miranda_RNAhybrid	-24	153	38	64	-26.9	0.633167	40	65	NM_133940.3	Fbxl14	mRNA	chr6:119479668-119483886	+	mmu:101358	F-box/LRR-repeat protein 14
mmu-miR-147-3p	Hus1	Miranda_RNAhybrid	-21.61	163	70	91	-26.7	0.765537	64	92	NM_008316.4	Hus1	mRNA	chr11:8993137-9011191	-	mmu:15574	Checkpoint protein HUS1
mmu-miR-147	Iba57	Miranda_RNAhy	-21.74	154	2651	2672	-27.5	0.547648	2647	2673	NM_183110.1	Iba57	mRNA	chr11:59155369-5916374	-	mmu:216792	Putative transferase CAF17

-3p		brid												5			homolog, mitochondrial
mmu-miR-147-3p	Zzef1	Miranda_RNAhybrid	-25.65	162	569	589	-30.8	0.139223	570	590	NM_001045536.2	Zzef1	mRNA	chr11:72796226-72927120	+	mmu:195018	Zinc finger ZZ-type and EF-hand domain-containing protein 1
mmu-miR-147-3p	Enthd2	Miranda_RNAhybrid	-28.81	173	289	309	-30	0.064411	291	310	NM_183137.2	Enthd2	mRNA	chr11:120090522-120098695	-	mmu:78777	AP-4 complex accessory subunit tepsin
mmu-miR-147-3p	Tspyl4	Miranda_RNAhybrid	-22.7	156	2251	2273	-28	0.430284	2241	2273	NM_030203.2	Tspyl4	mRNA	chr10:34297421-34301320	+	mmu:72480	Putative uncharacterized protein
mmu-miR-147-3p	Ncln	Miranda_RNAhybrid	-23.38	150	135	155	-26.2	0.370782	138	156	NM_134009.3	Ncln	mRNA	chr10:81486455-81496691	-	mmu:103425	Nicalin
mmu-miR-147-3p	Atp2b1	Miranda_RNAhybrid	-21.69	152	91	112	-25.8	0.849202	87	113	NM_026482.2	Atp2b1	miscRNA	chr10:98914359-99023295	+	mmu:67972	Plasma membrane calcium-transporting

																	ATPase 1
mmu-miR-147-3p	Cerk	Miranda_RNAhybrid	-22.74	150	548	570	-28.1	0.398344	551	570	NM_145475.4	Cerk	mRNA	chr15:86139129-86186336	-	mmu:223753	Ceramide kinase
mmu-miR-147-3p	Gxylt1	Miranda_RNAhybrid	-20.04	151	2629	2650	-26.8	0.856991	2619	2651	NM_001033275.4	Gxylt1	mRNA	chr15:93239735-93275336	-	mmu:223827	Glucoside xylosyltransferase 1
mmu-miR-147-3p	Zdhhc2	Miranda_RNAhybrid	-22.95	154	1538	1555	-28.6	0.280816	1538	1555	NM_178395.3	Zdhhc2	mRNA	chr8:40423002-40489592	+	mmu:70546	Palmitoyltransferase ZDHHC2
mmu-miR-147-3p	Sugp2	Miranda_RNAhybrid	-26.61	155	45	68	-32	0.163392	45	68	NM_172755.3	Sugp2	mRNA	chr8:70234549-70263105	+	mmu:234373	SURP and G-patch domain-containing protein 2
mmu-miR-147-3p	Foxl1	Miranda_RNAhybrid	-29.84	153	225	250	-33.9	0.022396	225	251	NM_008024.2	Foxl1	mRNA	chr8:121127685-121130644	+	mmu:14241	Forkhead box protein L1
mmu-miR-147	Slc7a5	Miranda_RNAhy	-23.22	155	396	418	-31.4	0.080776	393	418	NM_011404.3	Slc7a5	mRNA	chr8:121881146-1219076	-	mmu:20539	Putative uncharacterized protein

-3p		brid												86			
mmu-miR-147-3p	Tnik	Miranda_RNAhybrid	-22.89	151	2866	2884	-28.5	0.675151	2867	2885	NM_026910.1	Tnik	mRNA	chr3:28263214-28670585	+	mmu:665113	Tnik protein
mmu-miR-147-3p	Il21	Miranda_RNAhybrid	-24.91	150	1373	1398	-27.9	0.440249	1373	1398	NM_021782.3	Il21	mRNA	chr3:37222759-37232636	-	mmu:60505	Interleukin-21
mmu-miR-147-3p	Spry1	Miranda_RNAhybrid	-23.29	150	471	494	-27.9	0.177262	465	495	NM_011896.2	Spry1	mRNA	chr3:37639947-37644599	+	mmu:24063	Sprouty1
mmu-miR-147-3p	Arhgef11	Miranda_RNAhybrid	-23.38	158	441	463	-28.9	0.097504	445	463	NM_001003912.1	Arhgef11	mRNA	chr3:87617548-87738034	+	mmu:213498	Protein Arhgef11
mmu-miR-147-3p	Fam19a3	Miranda_RNAhybrid	-22.21	157	539	561	-31.7	0.139952	532	562	NM_183224.3	Fam19a3	mRNA	chr3:104767405-104774678	-	mmu:329731	Protein FAM19A3
mmu-miR-147-3p	Amph	Miranda_RNAhybrid	-21.01	154	2937	2959	-27.1	0.748858	2953	2977	NM_175007.2	Amph	mRNA	chr13:18948351-19150919	+	mmu:218038	Amphiphysin

mmu-miR-147-3p	Auh	Miranda_RNAhybrid	-24.72	166	246	268	-31.5	0.099566	243	269	NM_016709.2	Auh	mRNA	chr13:52907020-52929705	-	mmu:11992	Methylglutaconyl-CoA hydratase, mitochondrial
mmu-miR-147-3p	2010111I01Rik	Miranda_RNAhybrid	-25.41	159	3813	3832	-29.7	0.357533	3814	3832	NM_028079.2	2010111I01Rik	mRNA	chr13:62964893-63302877	+	mmu:72061	2010111I01Rik protein
mmu-miR-147-3p	Mre11a	Miranda_RNAhybrid	-24.64	162	2873	2893	-29.7	0.311972	2873	2893	NM_018736.2	Mre11a	mRNA	chr9:14790642-14837125	+	mmu:17535	Double-strand break repair protein MRE11A
mmu-miR-147-3p	AI593442	Miranda_RNAhybrid	-23.17	153	349	368	-28.6	0.557957	2650	2668	NM_178906.5	AI593442	mRNA	chr9:52673032-52679780	-	mmu:330941	Uncharacterized protein C11orf87 homolog
mmu-miR-147-3p	Hykk	Miranda_RNAhybrid	-20.39	150	1242	1267	-26.5	0.764182	663	682	NM_177351.4	Hykk	mRNA	chr9:54917290-54949924	+	mmu:235386	Hydroxylysine kinase
mmu-miR-147-3p	Myo1e	Miranda_RNAhybrid	-21.22	162	848	869	-26.3	0.412346	847	869	NM_181072.3	Myo1e	mRNA	chr9:70260427-70399766	+	mmu:71602	Unconventional myosin-Ie

mmu-miR-147-3p	Myo5a	Miranda_RNAhybrid	-23.31	167	2240	2261	-29.3	0.53025	2239	2262	NM_010864.2	Myo5a	mRNA	chr9:75071015-75223688	+	mmu:17918	Unconventional myosin-Va
mmu-miR-147-3p	Ctdspl	Miranda_RNAhybrid	-23.79	157	1877	1898	-30.5	0.233688	1883	1899	NM_133710.3	Ctdspl	mRNA	chr9:118929872-119044119	+	mmu:69274	CTD small phosphatase-like protein
mmu-miR-147-3p	Kdm2a	Miranda_RNAhybrid	-23.89	168	2168	2192	-30	0.239515	2171	2192	NM_001001984.2	Kdm2a	mRNA	chr19:4316144-4397077	-	mmu:225876	Lysine-specific demethylase 2A
mmu-miR-147-3p	9130011E15Rik	Miranda_RNAhybrid	-28.92	162	1181	1201	-34.6	0.014148	1180	1201	NM_198296.2	9130011E15Rik	mRNA	chr19:45817364-45998570	-	mmu:71617	UPF0668 protein C10orf76 homolog
mmu-miR-147-3p	Napa	Miranda_RNAhybrid	-20.2	151	1081	1100	-25.8	0.532808	1065	1100	NM_025898.3	Napa	mRNA	chr7:16098456-16113867	+	mmu:108124	Putative uncharacterized protein
mmu-miR-147-3p	Tmem145	Miranda_RNAhybrid	-28.01	154	288	310	-29	0.091361	290	311	NM_183311.2	Tmem145	mRNA	chr7:25305995-25316196	+	mmu:330485	Transmembrane protein 145
mmu-miR	Ctu1	Miranda_R	-22.81	164	323	344	-26.8	0.30123	309	345	NM_1455	Ctu1	mRN	chr7:43671976-4	+	mmu:233	Cytoplasmic tRNA

-147-3p		NAhybrid									82.1		A	3678300		189	2-thiolation protein 1
mmu-miR-147-3p	Akt1s1	Miranda_RNAhybrid	-20.14	160	139	161	-29.1	0.057305	135	161	NM_026270.4	Akt1s1	mRNA	chr7:44850007-44855421	+	mmu:67605	Proline-rich AKT1 substrate 1
mmu-miR-147-3p	Spcs2	Miranda_RNAhybrid	-21.78	151	1938	1963	-27.5	0.430499	1936	1963	NM_025668.3	Spcs2	mRNA	chr7:99837569-99858883	-	mmu:66624	Signal peptidase complex subunit 2
mmu-miR-147-3p	Stradb	Miranda_RNAhybrid	-20.24	154	229	250	-25.6	0.28619	217	251	NM_173155.1	Stradb	miscRNA	chr1:58973527-58995126	+	mmu:227154	STE20-related kinase adapter protein beta
mmu-miR-147-3p	Mgat5	Miranda_RNAhybrid	-21.75	157	2819	2844	-27	0.852337	2818	2844	NM_145128.3	Mgat5	mRNA	chr1:127204973-127488334	+	mmu:107895	Mannoside acetylglucosaminyltransferase 5
mmu-miR-147-3p	Lamc1	Miranda_RNAhybrid	-24.11	154	2307	2331	-28.3	0.384614	2308	2331	NM_010683.2	Lamc1	mRNA	chr1:153218922-153332786	-	mmu:226519	Laminin subunit gamma-1
mmu-miR-147-3p	Tmprss11f	Miranda_RNAhybrid	-25.76	161	1268	1287	-31.3	0.117673	1269	1288	NM_178730.3	Tmprss11f	mRNA	chr5:86521226-86632424	-	mmu:243083	Transmembrane protease serine 11F

mmu-miR-147-3p	Fgf5	Miranda_RNAhybrid	-27.49	152	801	820	-31.7	0.051013	800	821	NM_010203.5	Fgf5	mRNA	chr5:98254184-98277033	+	mmu:14176	Fibroblast growth factor 5
mmu-miR-147-3p	Lrrc8b	Miranda_RNAhybrid	-23.35	152	2960	2981	-29.1	0.432036	2959	2982	NM_001033550.2	Lrrc8b	mRNA	chr5:105415775-105486189	+	mmu:433926	Volume-regulated anion channel subunit LRRC8B
mmu-miR-147-3p	Rnft2	Miranda_RNAhybrid	-21.65	151	1231	1251	-27.2	0.581524	1234	1251	NM_172998.3	Rnft2	mRNA	chr5:118190734-118245111	-	mmu:269695	RING finger and transmembrane domain-containing protein 2
mmu-miR-147-3p	Ttyh3	Miranda_RNAhybrid	-26.94	154	80	104	-31.4	0.139434	80	105	NM_175274.4	Ttyh3	mRNA	chr5:140620577-140649031	-	mmu:78339	Protein tweety homolog 3
mmu-miR-147-3p	Pax9	Miranda_RNAhybrid	-21.05	151	240	262	-31	0.159477	230	263	NM_011041.2	Pax9	mRNA	chr12:56694844-56712824	+	mmu:18511	Paired box protein Pax-9
mmu-miR	Prkch	Miranda_R	-25.43	155	842	866	-31.3	0.041711	841	867	NM_0088	Prkch	mRN	chr12:73584796-	+	mmu:187	Protein kinase C eta

-147-3p		NAhybrid									56.3		A	73778185		55	type
mmu-miR-147-3p	Gphb5	Miranda_RNAhybrid	-22.7	150	125	150	-27.9	0.189245	125	151	NM_175644.3	Gphb5	mRNA	chr12:75411720-75415817	-	mmu:217674	Glycoprotein hormone beta 5
mmu-miR-147-3p	Psen1	Miranda_RNAhybrid	-24.25	153	1260	1283	-31.3	0.219413	1260	1283	NM_008943.2	Psen1	mRNA	chr12:83688145-83738446	+	mmu:19164	Presenilin-1
mmu-miR-147-3p	Ddx24	Miranda_RNAhybrid	-26.01	155	98	121	-30	0.00571	97	122	NM_020494.3	Ddx24	mRNA	chr12:103407976-103425867	-	mmu:27225	ATP-dependent RNA helicase DDX24
mmu-miR-147-3p	Zfp174	Miranda_RNAhybrid	-20.58	151	2070	2088	-26.2	0.859431	2315	2334	NM_001081217.1	Zfp174	mRNA	chr16:3847190-3872051	+	mmu:385674	Protein Zfp174
mmu-miR-147-3p	Ercc4	Miranda_RNAhybrid	-22.13	168	1964	1985	-27.6	0.631792	1964	1986	NM_015769.2	Ercc4	miscRNA	chr16:13109689-13149367	+	mmu:50505	DNA repair endonuclease XPF
mmu-miR-147-3p	Nrip1	Miranda_RNAhybrid	-20.79	159	1084	1106	-26.2	0.838916	1085	1107	NM_173440.2	Nrip1	mRNA	chr16:76287403-76324808	-	mmu:268903	Nuclear receptor-interacting protein 1

mmu-miR-147-3p	LOC108168308	Miranda_RNAhybrid	-21.75	160	1196	1217	-30.5	0.102116	1527	1559						
mmu-miR-147-3p	Rab11fip3	Miranda_RNAhybrid	-21.71	159	1711	1733	-27.6	0.345672	1709	1734	NM_153140.2	Rab11fip3	miscRNA	chr17:26024378-26069550	-	mmu:215445	Rab11 family-interacting protein 3
mmu-miR-147-3p	Ankrd12	Miranda_RNAhybrid	-24.28	158	412	435	-28.3	0.588237	2827	2848	NM_001025572.1	Ankrd12	mRNA	chr17:65965703-66031551	-	mmu:106585	Protein Ankrd12
mmu-miR-147-3p	Nacc2	Miranda_RNAhybrid	-21.34	153	2231	2253	-26.9	0.795461	2224	2253	NM_026495.3	Nacc2	mRNA	chr2:26055536-26092396	-	mmu:67991	Nucleus accumbens-associated protein 2
mmu-miR-147-3p	Lrrc8a	Miranda_RNAhybrid	-26.4	154	14	37	-32.9	0.035024	11	38	NM_177725.4	Lrrc8a	mRNA	chr2:30237769-30263790	+	mmu:241296	Volume-regulated anion channel subunit LRRC8A
mmu-miR-147-3p	Rims4	Miranda_RNAhybrid	-29.66	163	977	1001	-34	0.071921	978	1002	NM_183023.1	Rims4	mRNA	chr2:163863881-163918683	-	mmu:241770	Rims4 protein

mmu-miR-147-3p	Cyp24a1	Miranda_RNAhybrid	-20.57	155	720	742	-28	0.236355	720	743	NM_009996.3	Cyp24a1	mRNA	chr2:170482957-170497145	-	mmu:13081	Putative uncharacterized protein
mmu-miR-147-3p	Tnfaip8	Miranda_RNAhybrid	-20.07	151	1723	1742	-29.4	0.249543	1976	1995	NM_134131.2	Tnfaip8	mRNA	chr18:49979427-50093229	+	mmu:106869	Tumor necrosis factor alpha-induced protein 8
mmu-miR-147-3p	LOC108168395	Miranda_RNAhybrid	-26.02	168	1057	1078	-28.6	0.194703	1057	1079							
mmu-miR-98-5p	Pappa	Miranda_RNAhybrid	-22.79	165	4255	4280	-26.8	0.792268	4254	4281	NM_021362.1	Pappa	mRNA	chr4:65124174-65357509	+	mmu:18491	Pappalysin-1
mmu-miR-98-5p	Acot11	Miranda_RNAhybrid	-21.14	166	3677	3701	-28.9	0.330034	3676	3702	NM_025590.4	Acot11	mRNA	chr4:106744561-106799831	-	mmu:329910	Acyl-coenzyme A thioesterase 11
mmu-miR-98-5p	Svopl	Miranda_RNAhybrid	-22.02	162	866	888	-25.2	0.559112	868	889	NM_177200.4	Svopl	mRNA	chr6:37984752-38047012	-	mmu:320590	Putative transporter SVOPL

mmu-miR-98-5p	Tet3	Miranda_RNAhybrid	-20.26	154	2689	2713	-27.5	0.680474	3546	3570	NM_183138.2	Tet3	mRNA	chr6:83362373-83457242	-	mmu:194388	Methylcytosine dioxygenase TET3
mmu-miR-98-5p	Gm5878	Miranda_RNAhybrid	-20.88	164	875	896	-27.6	0.531513	400	430	NM_00103490 2.2	Gm5878	mRNA	chr6:85111416-85126094	-	mmu:545861	Protein Gm5878
mmu-miR-98-5p	Etnk1	Miranda_RNAhybrid	-22.63	155	3428	3451	-29	0.413679	3429	3452	NM_029250.2	Etnk1	mRNA	chr6:143167230-143208546	+	mmu:75320	Putative uncharacterized protein
mmu-miR-98-5p	Gm40814	Miranda_RNAhybrid	-22.73	165	100	122	-28.4	0.127641	99	122							
mmu-miR-98-5p	Sowaha	Miranda_RNAhybrid	-21.89	171	569	591	-25.6	0.515767	565	592	NM_183173.2	Sowaha	mRNA	chr11:53476578-53480195	-	mmu:237761	Ankrd43 protein
mmu-miR-98-5p	Il13	Miranda_RNAhybrid	-21.41	165	434	456	-26.9	0.122299	421	457	NM_008355.3	Il13	mRNA	chr11:53631323-53634702	-	mmu:16163	Interleukin-13
mmu-miR	Acsl6	Miranda_R	-20.85	155	390	413	-25.1	0.871055	380	414	NM_1448	Acsl6	mRN	chr11:54315297-	+	mmu:216	Long-chain acyl-CoA

-98-5p		NAhybrid									23.4		A	54364126		739	synthetase
mmu-miR-98-5p	Fnip1	Miranda_RNAhybrid	-24.37	176	1159	1180	-25.9	0.652079	1160	1181	NM_173753.4	Fnip1	mRNA	chr11:54438145-54516884	+	mmu:216742	Folliculin-interacting protein 1
mmu-miR-98-5p	Jmjd4	Miranda_RNAhybrid	-24.38	165	316	338	-27.1	0.495957	316	339	NM_178659.6	Jmjd4	mRNA	chr11:59450045-59458567	+	mmu:194952	JmjC domain-containing protein 4
mmu-miR-98-5p	LOC108167918	Miranda_RNAhybrid	-22.73	165	100	122	-28.4	0.136124	99	122							
mmu-miR-98-5p	Igf2bp1	Miranda_RNAhybrid	-25.12	172	4122	4143	-28.7	0.534799	4121	4144	NM_009951.4	Igf2bp1	mRNA	chr11:95957163-96005944	-	mmu:140486	Insulin-like growth factor 2 mRNA-binding protein 1
mmu-miR-98-5p	LOC108167806	Miranda_RNAhybrid	-22.73	165	100	122	-28.4	0.122471	99	122							
mmu-miR	LOC1081	Miranda_R	-22.73	165	157	179	-28.4	0.075538	156	179							

-98-5p	67809	NAhybrid															
mmu-miR-98-5p	Fam207a	Miranda_RNAhybrid	-21.35	150	1411	1434	-26.4	0.39134	1409	1435	NM_133998.3	Fam207a	mRNA	chr10:77486661-77516924	-	mmu:108707	Protein FAM207A
mmu-miR-98-5p	Cdc34	Miranda_RNAhybrid	-23.23	158	54	75	-25.9	0.065382	59	76	NM_177613.2	Cdc34	mRNA	chr10:79682203-79688398	+	mmu:216150	Ubiquitin-conjugating enzyme E2 R1
mmu-miR-98-5p	Fgd6	Miranda_RNAhybrid	-22.32	161	507	528	-25.2	0.854535	494	529	NM_053072.3	Fgd6	mRNA	chr10:94036001-94145339	+	mmu:13998	Fgd6 protein
mmu-miR-98-5p	Hmga2	Miranda_RNAhybrid	-21.83	164	8	28	-25.3	0.79013	8	29	NM_010441.2	Hmga2	mRNA	chr10:120361275-120476935	-	mmu:15364	High mobility group protein HMGI-C
mmu-miR-98-5p	Kcnv1	Miranda_RNAhybrid	-21.23	164	3031	3055	-25.3	0.997449	3029	3055	NM_026200.3	Kcnv1	mRNA	chr15:45096086-45116490	-	mmu:67498	Potassium voltage-gated channel subfamily V member 1
mmu-miR	Pced1b	Miranda_R	-22.63	165	226	247	-26.5	0.040552	225	247	NM_1722	Pced1b	mRN	chr15:97247412-	+	mmu:239	PC-esterase domain-con

-98-5p		NAhybrid									93.4		A	97385693		647	taining protein 1B
mmu-miR-98-5p	Dhh	Miranda_RNAhybrid	-20.03	170	2059	2079	-25.1	0.796653	2058	2079	NM_007857.4	Dhh	mRNA	chr15:98893027-98895957	-	mmu:13363	Desert hedgehog protein
mmu-miR-98-5p	Letmd1	Miranda_RNAhybrid	-21.81	164	1064	1086	-25.6	0.418249	1063	1086	NM_134093.2	Letmd1	mRNA	chr15:100469018-100479164	+	mmu:68614	LETM1 domain-containing protein 1
mmu-miR-98-5p	Cbx5	Miranda_RNAhybrid	-20.44	159	6659	6681	-28.2	0.700872	1036	1067	NM_007626.3	Cbx5	mRNA	chr15:103191678-103215362	-	mmu:12419	Chromobox protein homolog 5
mmu-miR-98-5p	Hsh2d	Miranda_RNAhybrid	-20.39	167	138	161	-25.4	0.028911	137	161	NM_197944.1	Hsh2d	mRNA	chr8:72189668-72200958	+	mmu:209488	Hematopoietic SH2 domain-containing protein
mmu-miR-98-5p	Nkd1	Miranda_RNAhybrid	-21.62	158	359	382	-27	0.481183	363	382	NM_027280.3	Nkd1	mRNA	chr8:88521344-88594887	+	mmu:93960	Protein naked cuticle homolog 1
mmu-miR-98-	Nod2	Miranda_RNAhy	-20.27	169	1362	1385	-25.2	0.536581	1361	1385	NM_145857.2	Nod2	mRNA	chr8:88647314-88684464	+	mmu:257632	CARD15 short isoform

5p		brid															
mmu-miR-98-5p	Elmo3	Miranda_RNAhybrid	-22.82	170	204	225	-30.3	0.022372	200	225	NM_172760.3	Elmo3	mRNA	chr8:105305601-105310623	+	mmu:234683	Elmo3 protein
mmu-miR-98-5p	Clcn5	Miranda_RNAhybrid	-24	171	4145	4167	-25.5	0.939365	4145	4168	NM_016691.4	Clcn5	mRNA	chrx:7155160-7276896	-	mmu:12728	Chloride channel protein
mmu-miR-98-5p	Gucy2f	Miranda_RNAhybrid	-21.92	157	3453	3477	-25.7	0.937759	3451	3477	NM_001007576.2	Gucy2f	mRNA	chrx:142083290-142197276	-	mmu:245650	Retinal guanylyl cyclase 2
mmu-miR-98-5p	Klf8	Miranda_RNAhybrid	-23.61	176	1479	1500	-26.5	0.788871	1478	1500	NM_173780.4	Klf8	mRNA	chrx:153237422-153397886	+	mmu:245671	Krueppel-like factor 8
mmu-miR-98-5p	Gja8	Miranda_RNAhybrid	-21.23	167	3346	3368	-27.3	0.697721	142	167	NM_008123.3	Gja8	mRNA	chr3:96913566-96926051	-	mmu:14616	Gap junction alpha-8 protein
mmu-miR-98-5p	Cyb561d1	Miranda_RNAhybrid	-21.93	168	1205	1226	-26.2	0.701613	1210	1227	NM_028061.1	Cyb561d1	mRNA	chr3:108195771-108202126	-	mmu:72023	Cytochrome b561 domain-containing

																	protein 1
mmu-miR-98-5p	LOC108168067	Miranda_RNAhybrid	-22.73	165	100	122	-28.4	0.036702	99	122							
mmu-miR-98-5p	Gm41035	Miranda_RNAhybrid	-22.73	165	157	179	-28.4	0.129486	156	179							
mmu-miR-98-5p	Vstm5	Miranda_RNAhybrid	-20.65	164	395	415	-25.1	0.568259	387	416	NM_026955.2	Vstm5	miscRNA	chr9:15238923-15259421	+	mmu:69137	V-set and transmembrane domain-containing protein 5
mmu-miR-98-5p	Rnf214	Miranda_RNAhybrid	-21.44	162	5388	5411	-25.8	0.963327	5388	5411	NM_178709.4	Rnf214	mRNA	chr9:45856769-45906435	-	mmu:235315	RING finger protein 214
mmu-miR-98-5p	Rdx	Miranda_RNAhybrid	-20.6	167	962	983	-25.6	0.63668	951	984	NM_009041.3	Rdx	mRNA	chr9:52047150-52088738	+	mmu:19684	Radixin
mmu-miR	1700017B	Miranda_R	-21.81	159	263	286	-25.3	0.301013	267	287	NM_0288	1700017B	mRN	chr9:57253117-5	-	mmu:742	Uncharacterized protein

-98-5p	05Rik	NAhybrid									20.2	05Rik	A	7262410		11	C15orf39 homolog
mmu-miR-98-5p	Tmod2	Miranda_RNAhybrid	-23.37	163	5792	5816	-27	0.874412	5791	5817	NM_016711.3	Tmod2	mRNA	chr9:75565621-75610330	-	mmu:50876	Tropomodulin-2
mmu-miR-98-5p	Prr23a3	Miranda_RNAhybrid	-21.67	170	235	257	-25.4	0.279247	240	257							
mmu-miR-98-5p	Tusc2	Miranda_RNAhybrid	-21.85	159	473	497	-26.3	0.28811	472	498	NM_019742.4	Tusc2	mRNA	chr9:107563255-107566108	+	mmu:80385	Tumor suppressor candidate 2
mmu-miR-98-5p	Vps13a	Miranda_RNAhybrid	-21.03	166	880	900	-25.7	0.732931	870	901	NM_173028.4	Vps13a	mRNA	chr19:16615366-16780933	-	mmu:271564	Putative uncharacterized protein Vps13a 5' variant II
mmu-miR-98-5p	Tmem2	Miranda_RNAhybrid	-20.63	162	1545	1566	-26.7	0.4109	1542	1567	NM_031997.4	Tmem2	mRNA	chr19:21778340-21858360	+	mmu:83921	Transmembrane protein 2
mmu-miR-98-	Kcnk6	Miranda_RNAhy	-21.54	159	2885	2904	-25.2	0.818737	2888	2905	NM_0010 3352	Kcnk6	mRNA	chr7:29221928-29232522	-	mmu:52150	Kcnk6 protein

5p		brid									5.3						
mmu-miR-98-5p	LOC108167415	Miranda_RNAhybrid	-22.73	165	100	122	-28.4	0.08787	99	122							
mmu-miR-98-5p	Sec1	Miranda_RNAhybrid	-20.17	159	496	518	-25.2	0.281737	419	438	NM_019934.2	Sec1	mRNA	chr7:45677686-45684247	-	mmu:56546	Galactoside 2-alpha-L-fucosyltransferase 3
mmu-miR-98-5p	E030018B13Rik	Miranda_RNAhybrid	-22.9	160	916	934	-29.3	0.054191	915	935	NM_00125631 1.1	E030018B13Rik	mRNA	chr7:63919164-63921029	+	mmu:381994	Protein E030018B13Rik
mmu-miR-98-5p	LOC108167434	Miranda_RNAhybrid	-22.73	165	100	122	-28.4	0.136124	99	122							
mmu-miR-98-5p	Clpb	Miranda_RNAhybrid	-22.19	165	908	929	-26.5	0.594319	912	930	NM_009191.3	Clpb	mRNA	chr7:101721982-101790721	+	mmu:20480	Caseinolytic peptidase B protein homolog
mmu-miR-98-5p	Eef2k	Miranda_RNAhybrid	-22.66	169	623	645	-25.3	0.866593	624	645	NM_007908.4	Eef2k	mRNA	chr7:120842829-120907217	+	mmu:13631	Eukaryotic elongation factor 2 kinase

mmu-miR-98-5p	Mrpl15	Miranda_RNAhybrid	-20.02	153	1090	1111	-26	0.714289	2452	2478	NM_025300.4	Mrpl15	miscRNA	chr1:4773198-4785726	-	mmu:27395	Mitochondrial ribosomal protein L15, isoform CRA_a
mmu-miR-98-5p	Fam126b	Miranda_RNAhybrid	-20.99	151	3947	3969	-25.6	0.953668	6215	6241	NM_172513.3	Fam126b	mRNA	chr1:58522806-58586154	-	mmu:213056	Protein FAM126B
mmu-miR-98-5p	Ccdc93	Miranda_RNAhybrid	-22.26	160	3028	3049	-25.2	0.941303	3028	3049	NM_029955.3	Ccdc93	mRNA	chr1:121431067-121506460	+	mmu:70829	Coiled-coil domain-containing protein 93
mmu-miR-98-5p	LOC108167725	Miranda_RNAhybrid	-22.73	165	100	122	-28.4	0.129609	99	122							
mmu-miR-98-5p	Fasl	Miranda_RNAhybrid	-22.97	167	350	371	-26.6	0.178048	350	372	NM_010177.4	Fasl	mRNA	chr1:161780691-161788495	-	mmu:14103	Fas ligand
mmu-miR-98-5p	LOC105244034	Miranda_RNAhybrid	-22.73	165	157	179	-28.4	0.134649	156	179							

mmu-miR-98-5p	Gm39743	Miranda_RNAhybrid	-22.73	165	289	311	-28.4	0.118531	288	311						
mmu-miR-98-5p	Gm21759	Miranda_RNAhybrid	-26.29	172	77	96	-27.8	0.071636	77	97						
mmu-miR-98-5p	Gm42102	Miranda_RNAhybrid	-22.73	165	100	122	-28.4	0.11927	99	122						
mmu-miR-98-5p	Fras1	Miranda_RNAhybrid	-22.7	165	145	169	-26.3	0.626215	147	170	NM_175473.3	Fras1	mRNA	chr5:96373819-96784733	+	mmu:231470	Extracellular matrix protein FRAS1
mmu-miR-98-5p	LOC108168003	Miranda_RNAhybrid	-22.73	165	100	122	-28.4	0.124318	99	122						
mmu-miR-98-5p	Grin2a	Miranda_RNAhybrid	-21.08	155	2938	2963	-25.1	0.928136	2938	2963	NM_008170.2	Grin2a	mRNA	chr16:9621275-9995132	-	mmu:14811	Glutamate receptor ionotropic, NMDA 2A
mmu-miR	Rabl3	Miranda_R	-21.34	156	95	116	-25	0.507652	94	117	NM_0262	Rabl3	mRN	chr16:37539894-	+	mmu:676	Rab-like protein 3

-98-5p		NAhybrid									97.1		A	37572385		57	
mmu-miR-98-5p	Gm41476	Miranda_RNAhybrid	-22.73	165	100	122	-28.4	0.066718	99	122							
mmu-miR-98-5p	4930488N24Rik	Miranda_RNAhybrid	-23.17	158	328	352	-25.1	0.301515	330	353	XR_397312.1	4930488N24Rik	mRNA	chr17:14101817-14107866	-	mmu:75011	RIKEN cDNA 4930488N24 gene
mmu-miR-98-5p	Wdr27	Miranda_RNAhybrid	-23.42	156	88	107	-26.9	0.28074	87	107	NM_175173.3	Wdr27	mRNA	chr17:14784829-14943151	-	mmu:71682	WD repeat-containing protein 27
mmu-miR-98-5p	Crb3	Miranda_RNAhybrid	-23.95	171	152	174	-29.4	0.203187	151	174	NM_177638.4	Crb3	mRNA	chr17:57061627-57065920	+	mmu:224912	Protein crumbs homolog 3
mmu-miR-98-5p	Gm37416	Miranda_RNAhybrid	-22.73	165	363	385	-28.4	0.161786	362	385							
mmu-miR-98-5p	Tbc1d13	Miranda_RNAhybrid	-21.83	155	1186	1208	-25	0.735875	1186	1208	NM_146252.2	Tbc1d13	mRNA	chr2:30142536-30152012	+	mmu:70296	TBC1 domain family member 13

mmu-miR-98-5p	Pbx3	Miranda_RNAhybrid	-22.34	165	858	879	-25	0.549198	860	879	NM_016768.2	Pbx3	mRNA	chr2:34171457-34371692	-	mmu:18516	Pre-B-cell leukemia transcription factor 3
mmu-miR-98-5p	Katnbl1	Miranda_RNAhybrid	-21.23	161	1053	1072	-25.4	0.582799	1053	1072	NM_024254.3	Katnbl1	mRNA	chr2:112379211-112414237	+	mmu:72425	Putative uncharacterized protein
mmu-miR-98-5p	Trp53bp1	Miranda_RNAhybrid	-20.34	168	3080	3101	-25.6	0.807987	3074	3101	NM_013735.4	Trp53bp1	mRNA	chr2:121194835-121252027	-	mmu:27223	Tumor suppressor p53-binding protein 1
mmu-miR-98-5p	Grpel2	Miranda_RNAhybrid	-22.35	172	2436	2457	-27	0.55859	2436	2457	NM_021296.2	Grpel2	miscRNA	chr18:61712424-61726360	-	mmu:17714	GrpE protein homolog 2, mitochondrial
mmu-miR-98-5p	Ptpn2	Miranda_RNAhybrid	-23.97	165	3401	3422	-25.3	0.972319	3405	3423	NM_008977.3	Ptpn2	mRNA	chr18:67665501-67724758	-	mmu:19255	Tyrosine-protein phosphatase non-receptor type 2
mmu-miR-98-	LOC1081 6837	Miranda_RNAhy	-22.73	165	100	122	-28.4	0.148258	99	122							

5p	6	brid															
mmu-miR-10a-5p	Ankrd6	Miranda_RNAhybrid	-22.41	153	1029	1055	-25.2	0.825686	1031	1056	NM_080471.3	Ankrd6	mRNA	chr4:32804027-32864810	-	mmu:140577	Ankyrin repeat domain-containing protein 6
mmu-miR-10a-5p	Tmod1	Miranda_RNAhybrid	-23.72	154	1455	1478	-25.3	0.729402	1456	1479	NM_021883.2	Tmod1	mRNA	chr4:46038940-46104015	+	mmu:21916	Tropomodulin-1
mmu-miR-10a-5p	Nr4a3	Miranda_RNAhybrid	-25.38	161	1046	1068	-28.7	0.441534	1036	1069	NM_015743.2	Nr4a3	mRNA	chr4:48044889-48086447	+	mmu:18124	Nr4a3 protein
mmu-miR-10a-5p	Cer1	Miranda_RNAhybrid	-20.21	160	208	232	-25.1	0.489133	207	232	NM_009887.2	Cer1	mRNA	chr4:82881751-82885151	-	mmu:12622	Cerberus 1 homolog (Xenopus laevis)
mmu-miR-10a-5p	Faf1	Miranda_RNAhybrid	-23.84	167	1962	1987	-27.1	0.528391	1964	1987	NM_007983.2	Faf1	mRNA	chr4:109676627-109963960	+	mmu:14084	FAS-associated factor 1
mmu-miR-10a	Slc5a9	Miranda_RNAhy	-22.91	163	1690	1714	-26.2	0.68854	1690	1715	NM_145551.4	Slc5a9	miscRN	chr4:111885528-1119027	-	mmu:230612	Sodium/glucose cotransport

-5p		brid											A	83			er 4
mmu-miR-10a-5p	D830031N03Rik	Miranda_RNAhybrid	-23.06	164	2357	2383	-28	0.618101	2357	2383	NM_00116791 8.1	D830031N03Rik	mRNA	chr4:123403601-123411911	-	mmu:442834	Uncharacterized protein KIAA0754
mmu-miR-10a-5p	Gm13083	Miranda_RNAhybrid	-26.26	165	656	678	-28.5	0.141652	659	679	NM_00112632 4.1	Gm13083	mRNA	chr4:143615060-143618738	+	mmu:279185	Protein Gm13083
mmu-miR-10a-5p	Agtrap	Miranda_RNAhybrid	-22.61	152	2808	2831	-26.5	0.805866	2800	2832	NM_009642.4	Agtrap	mRNA	chr4:148077061-148088064	-	mmu:11610	Type-1 angiotensin II receptor-associated protein
mmu-miR-10a-5p	Vwa1	Miranda_RNAhybrid	-21.77	168	1062	1084	-26.2	0.623187	1059	1084	NM_147776.4	Vwa1	mRNA	chr4:155769300-155772798	-	mmu:246228	von Willebrand factor A domain-containing protein 1
mmu-miR-10a-5p	Nxph1	Miranda_RNAhybrid	-24.96	160	602	626	-26.2	0.996515	603	627	NM_008751.5	Nxph1	mRNA	chr6:8947788-9259863	+	mmu:18231	Neurexophilin

mmu-miR-10a-5p	Irf5	Miranda_RNAhybrid	-25.03	156	75	99	-26.3	0.181801	75	99	NM_012057.4	Irf5	mRNA	chr6:29529243-29537320	+	mmu:27056	Interferon regulatory factor 5 bone marrow variant
mmu-miR-10a-5p	Svopl	Miranda_RNAhybrid	-20.92	163	201	222	-29.5	0.16962	196	222	NM_177200.4	Svopl	mRNA	chr6:37984752-38047012	-	mmu:320590	Putative transporter SVOPL
mmu-miR-10a-5p	Adck2	Miranda_RNAhybrid	-20.73	159	824	845	-28.7	0.184261	805	846	NM_178873.3	Adck2	mRNA	chr6:39573876-39588769	+	mmu:57869	Uncharacterized aarF domain-containing protein kinase 2
mmu-miR-10a-5p	Arhgap25	Miranda_RNAhybrid	-21.74	156	75	97	-25.8	0.56515	67	98	NM_175476.4	Arhgap25	miscRNA	chr6:87458545-87496327	-	mmu:232201	Rho GTPase activating protein 25
mmu-miR-10a-5p	Prrt3	Miranda_RNAhybrid	-24.33	154	209	233	-25.2	0.367342	199	234	NM_172487.4	Prrt3	mRNA	chr6:113493638-113501843	-	mmu:210673	Proline-rich transmembrane protein 3, isoform CRA_b

mmu-miR-10a-5p	Vgll4	Miranda_RNAhybrid	-22.94	153	1407	1429	-26.7	0.47374	1405	1430	NM_177683.2	Vgll4	mRNA	chr6:114860621-114901036	-	mmu:232334	Transcription cofactor vestigial-like protein 4
mmu-miR-10a-5p	Syn2	Miranda_RNAhybrid	-21.74	160	1888	1913	-27.1	0.557856	1885	1914	NM_013681.3	Syn2	mRNA	chr6:115134902-115276563	+	mmu:20965	Synapsin-2
mmu-miR-10a-5p	Tmcc1	Miranda_RNAhybrid	-25.05	162	1079	1101	-28.4	0.527493	1087	1102	NM_177412.1	Tmcc1	mRNA	chr6:116018611-116193373	-	mmu:330401	Transmembrane and coiled-coil domains protein 1
mmu-miR-10a-5p	Cracr2a	Miranda_RNAhybrid	-22.59	153	893	917	-26.6	0.878015	894	918	NM_001033464.3	Cracr2a	miscRNA	chr6:127594740-127667664	+	mmu:381812	EF-hand calcium-binding domain-containing protein 4B
mmu-miR-10a-5p	Ascc2	Miranda_RNAhybrid	-22.73	155	1133	1158	-25.4	0.867102	1138	1159	NM_029291.1	Ascc2	mRNA	chr11:4637698-4685699	+	mmu:75452	Putative uncharacterized protein
mmu-miR-10a	Lcp2	Miranda_RNAhy	-26.23	174	467	489	-27.5	0.370749	468	490	NM_010696.3	Lcp2	mRNA	chr11:34047201-3409228	+	mmu:16822	Lymphocyte cytosolic protein 2

-5p		brid												0			
mmu-miR-10a-5p	Cyfip2	Miranda_RNAhybrid	-21.08	150	992	1011	-25	0.894121	997	1012	NM_133769.3	Cyfip2	mRNA	chr11:46193855-46312364	-	mmu:76884	Cytoplasmic FMR1-interacting protein 2
mmu-miR-10a-5p	Shroom1	Miranda_RNAhybrid	-22.27	163	359	380	-29.8	0.047559	351	380	NM_027917.3	Shroom1	mRNA	chr11:53457205-53467766	+	mmu:71774	Shroom family member 1, isoform CRA_a
mmu-miR-10a-5p	Slc2a4	Miranda_RNAhybrid	-21.9	150	958	978	-25.1	0.589607	952	978	NM_009204.2	Slc2a4	mRNA	chr11:69942286-69948190	-	mmu:20528	Solute carrier family 2, facilitated glucose transporter member 4
mmu-miR-10a-5p	Ccdc92b	Miranda_RNAhybrid	-21.93	157	1723	1745	-27.3	0.633664	1721	1746							
mmu-miR-10a-5p	Mettl16	Miranda_RNAhybrid	-21.22	153	3532	3558	-25.5	0.997327	3530	3558	NM_026197.3	Mettl16	mRNA	chr11:74770837-74825823	+	mmu:67493	Methyltransferase-like protein 16

mmu-miR-10a-5p	Mmd	Miranda_RNAhybrid	-22.03	152	1196	1220	-29.2	0.225849	1193	1220	NM_026178.2	Mmd	mRNA	chr11:90249297-90251618	+	mmu:67468	Putative uncharacterized protein
mmu-miR-10a-5p	Smarce1	Miranda_RNAhybrid	-21.88	151	439	459	-25.5	0.497762	438	460	NM_020618.4	Smarce1	mRNA	chr11:99209047-99231339	-	mmu:57376	Smarce1 variant 3
mmu-miR-10a-5p	Zfp385c	Miranda_RNAhybrid	-25.55	156	526	545	-27.9	0.494522	525	546	NM_177790.4	Zfp385c	mRNA	chr11:100627543-100692450	-	mmu:278304	Zinc finger protein 385C
mmu-miR-10a-5p	Mapt	Miranda_RNAhybrid	-21.53	153	631	653	-25.2	0.95993	628	654	NM_010838.4	Mapt	mRNA	chr11:104231327-104332090	+	mmu:17762	Microtubule-associated protein tau
mmu-miR-10a-5p	Limd2	Miranda_RNAhybrid	-25.07	151	843	873	-28.9	0.333068	842	873	NM_172397.3	Limd2	mRNA	chr11:106158035-106159965	-	mmu:67803	LIM domain-containing protein 2
mmu-miR-10a-5p	Prkca	Miranda_RNAhybrid	-24.17	155	2587	2608	-26.9	0.911773	2586	2608	NM_011101.3	Prkca	mRNA	chr11:107933387-108343888	-	mmu:18750	Protein kinase C alpha type

mmu-miR-10a-5p	Map2k6	Miranda_RNAhybrid	-28.04	168	31	55	-29.7	0.338874	33	56	NM_011943.2	Map2k6	mRNA	chr11:110399103-110516201	+	mmu:26399	Dual-specificity mitogen-activated protein kinase kinase 6
mmu-miR-10a-5p	Nploc4	Miranda_RNAhybrid	-20.07	162	1268	1291	-25.2	0.893754	1267	1291	NM_199469.2	Nploc4	mRNA	chr11:120379798-120437700	-	mmu:217365	Nuclear protein localization protein 4 homolog
mmu-miR-10a-5p	Slc2a12	Miranda_RNAhybrid	-23.29	158	25	47	-27	0.466585	23	47	NM_178934.4	Slc2a12	mRNA	chr10:22612730-22703183	+	mmu:353169	Solute carrier family 2 (Facilitated glucose transporter), member 12
mmu-miR-10a-5p	C2cd4c	Miranda_RNAhybrid	-24.47	157	3971	3993	-26.7	0.895855	3970	3994	NM_198614.3	C2cd4c	mRNA	chr10:79606854-79614018	-	mmu:237397	C2 calcium-dependent domain-containing protein 4C

mmu-miR-10a-5p	Gna11	Miranda_RNAhybrid	-20.28	150	544	566	-25.2	0.822181	1315	1341	NM_010301.3	Gna11	mRNA	chr10:81528732-81545046	-	mmu:14672	Guanine nucleotide-binding protein subunit alpha-11
mmu-miR-10a-5p	Zfp433	Miranda_RNAhybrid	-22.15	165	1306	1325	-25.2	0.860484	1305	1325	NM_001243067.1	Zfp433	mRNA	chr10:81704192-81721976	+	mmu:73610	Protein Zfp433
mmu-miR-10a-5p	Gm32687	Miranda_RNAhybrid	-22.15	165	1306	1325	-25.2	0.858336	1305	1325							
mmu-miR-10a-5p	Gm33378	Miranda_RNAhybrid	-22.15	165	1304	1323	-25.2	0.904552	1303	1323							
mmu-miR-10a-5p	Vezt	Miranda_RNAhybrid	-24.21	159	3428	3450	-25.8	0.998613	3420	3451	NM_172538.4	Vezt	mRNA	chr10:93961522-94035799	-	mmu:215008	Vezatin
mmu-miR-10a-5p	Ankrd33b	Miranda_RNAhybrid	-24.97	153	995	1019	-26.2	0.9502	996	1020	NM_027496.3	Ankrd33b	mRNA	chr15:31291479-31367759	-	mmu:67434	Ankyrin repeat domain-containing

																	protein 33B
mmu-miR-10a-5p	Kcnv1	Miranda_RNAhybrid	-23.85	164	12575	12597	-28.2	0.939758	12571	12598	NM_026200.3	Kcnv1	mRNA	chr15:45096086-45116490	-	mmu:67498	Potassium voltage-gated channel subfamily V member 1
mmu-miR-10a-5p	Trappc9	Miranda_RNAhybrid	-22.89	154	1083	1105	-25.1	0.751305	1066	1106	NM_180662.2	Trappc9	mRNA	chr15:72589620-73055812	-	mmu:76510	Trafficking protein particle complex subunit 9
mmu-miR-10a-5p	1810041L15Rik	Miranda_RNAhybrid	-26.22	155	173	200	-29.2	0.55778	173	201	NM_001163145.1	1810041L15Rik	mRNA	chr15:84379203-84446666	-	mmu:72301	Protein 1810041L15Rik
mmu-miR-10a-5p	Ttll8	Miranda_RNAhybrid	-24.22	161	276	297	-27.1	0.421372	276	298	NM_172818.3	Ttll8	mRNA	chr15:88917458-88955642	-	mmu:239591	Protein monoglycylase TTLL8
mmu-miR-10a-5p	Slc38a2	Miranda_RNAhybrid	-22.09	155	903	925	-26.7	0.69958	904	925	NM_175121.3	Slc38a2	mRNA	chr15:96687392-96699724	-	mmu:67760	Putative uncharacterized protein
mmu-miR	Insr	Miranda_R	-24.4	166	772	794	-26	0.937264	773	795	NM_0105	Insr	mRN	chr8:3150922-32	-	mmu:163	Insulin receptor

-10a -5p		NAhy brid									68.2		A	79649		37	isoform B
mmu -miR -10a -5p	Ppp 1r3b	Miran da_R NAhy brid	-23. 47	155	674	695	-26.4	0.804 762	666	696	NM_ 1777 41.3	Ppp 1r3b	m RN A	chr8:353 75667-3 5388139	+	mmu :244 416	Protein phosphatas e 1 regulatory subunit 3B
mmu -miR -10a -5p	Nfix	Miran da_R NAhy brid	-22. 22	157	1803	1829	-25.9	0.912 115	1793	1829	NM_ 0109 06.3	Nfix	m RN A	chr8:847 04717-8 4780806	-	mmu :180 32	Nuclear factor 1 X-type
mmu -miR -10a -5p	Nfat 5	Miran da_R NAhy brid	-22. 45	161	1178	1200	-26.7	0.968 588	1176	1200	NM_ 1339 57.3	Nfat 5	m RN A	chr8:107 331084- 1073744 74	+	mmu :544 46	Nuclear factor of-activated T-cells 5
mmu -miR -10a -5p	Disc 1	Miran da_R NAhy brid	-22. 66	156	156	178	-27	0.748 996	152	179	NM_ 1748 54.2	Disc 1	m RN A	chr8:125 054195- 1252611 51	+	mmu :244 667	Disrupted in schizophreni a 1 homolog
mmu -miR -10a -5p	Myc s	Miran da_R NAhy brid	-24. 43	162	243	268	-28.3	0.127 982	244	269	NM_ 0108 50.2	Myc s	m RN A	chrx:546 6904-54 69265	-	mmu :178 70	Protein S-Myc
mmu -miR -10a	Atp7 a	Miran da_R NAhy	-24	165	755	777	-26.5	0.817 899	759	777	NM_ 0097 26.5	Atp7 a	m RN A	chrx:106 027224- 1061281	+	mmu :119 77	Copper-tran sporting ATPase 1

-5p		brid												60			
mmu-miR-10a-5p	Ndufb5	Miranda_RNAhybrid	-29.19	174	149	171	-32.8	0.004626	148	172	NM_025316.2	Ndufb5	mRNA	chr3:32737107-32751236	+	mmu:66046	NADH dehydrogenase [ubiquinone] 1 beta subcomplex subunit 5, mitochondrial
mmu-miR-10a-5p	E130311K13Rik	Miranda_RNAhybrid	-26.14	163	210	232	-30.7	0.029636	202	233	NM_177856.4	E130311K13Rik	mRNA	chr3:63914696-63929385	-	mmu:329659	Protein C3orf33 homolog
mmu-miR-10a-5p	Tmigd3	Miranda_RNAhybrid	-21.57	151	696	716	-25	0.586638	700	716							
mmu-miR-10a-5p	Adora3	Miranda_RNAhybrid	-21.57	151	696	716	-25	0.586638	700	716	NM_027025.4	Adora3	mRNA	chr3:105904421-105908928	+	mmu:11542	Adenosine receptor A3
mmu-miR-10a	Arsj	Miranda_RNAhy	-22.23	155	325	350	-26.2	0.371378	327	351	NM_173451.3	Arsj	mRNA	chr3:126363852-1264403	+	mmu:271970	Arylsulfatase J

-5p		brid												74			
mmu-miR-10a-5p	Tnni3k	Miranda_RNAhybrid	-25.47	171	135	157	-29.7	0.025451	133	158	NM_177066.5	Tnni3k	mRNA	chr3:154792779-155055402	-	mmu:435766	Serine/threonine-protein kinase TNNI3K
mmu-miR-10a-5p	Negr1	Miranda_RNAhybrid	-23.49	161	5674	5696	-27	0.893983	5674	5697	NM_177274.4	Negr1	mRNA	chr3:156561799-157228495	+	mmu:320840	Neuronal growth regulator 1
mmu-miR-10a-5p	E2f3	Miranda_RNAhybrid	-23.45	157	3212	3231	-25.8	0.883688	3004	3037	NM_010093.3	E2f3	mRNA	chr13:29906575-29984391	-	mmu:13557	E2F transcription factor 3
mmu-miR-10a-5p	Ssr1	Miranda_RNAhybrid	-26.36	150	5481	5503	-26.5	0.976044	5481	5504	NM_025965.3	Ssr1	mRNA	chr13:37971401-37994190	-	mmu:107513	Putative uncharacterized protein
mmu-miR-10a-5p	Jarid2	Miranda_RNAhybrid	-21.78	162	1163	1186	-25.5	0.704618	1163	1186	NM_021878.3	Jarid2	mRNA	chr13:44729535-44921638	+	mmu:16468	Protein Jumonji
mmu-miR-10a-5p	Id4	Miranda_RNAhybrid	-21.57	151	989	1010	-26	0.449168	989	1010	NM_031166.2	Id4	mRNA	chr13:48261427-48264036	+	mmu:15904	DNA-binding protein inhibitor ID-4

mmu-miR-10a-5p	2010111I01Rik	Miranda_RNAhybrid	-21.42	151	1375	1395	-26.4	0.865974	1376	1396	NM_028079.2	2010111I01Rik	mRNA	chr13:62964893-63302877	+	mmu:72061	2010111I01Rik protein
mmu-miR-10a-5p	Zfp367	Miranda_RNAhybrid	-24.77	160	209	236	-27.1	0.561023	210	237	NM_175494.4	Zfp367	mRNA	chr13:64133025-64152382	-	mmu:238673	Zinc finger protein 367
mmu-miR-10a-5p	Zfp65	Miranda_RNAhybrid	-23.4	157	1559	1580	-26.1	0.782461	1561	1580	NM_145622.2	Zfp65	mRNA	chr13:67705306-67729173	-	mmu:235907	Protein Zfp65
mmu-miR-10a-5p	Slc6a19	Miranda_RNAhybrid	-21.56	158	1117	1138	-26.6	0.568953	1114	1139	NM_028878.3	Slc6a19	mRNA	chr13:73679745-73692905	-	mmu:74338	Sodium-dependent neutral amino acid transporter B(0)AT1
mmu-miR-10a-5p	Cdk7	Miranda_RNAhybrid	-23.98	176	1901	1923	-26.9	0.921474	1901	1923	NM_009874.3	Cdk7	mRNA	chr13:100697024-100730942	-	mmu:12572	Cyclin-dependent kinase 7 (Homolog of Xenopus MO15 cdk-activating kinase),

																	isoform CRA_a
mmu-miR-10a-5p	Zfp26	Miranda_RNAhybrid	-23.92	164	5000	5022	-28.4	0.821215	4999	5023	NM_011753.3	Zfp26	mRNA	chr9:20433181-20460169	-	mmu:22688	Zinc finger protein 26
mmu-miR-10a-5p	Eepd1	Miranda_RNAhybrid	-23.35	157	41	63	-26	0.192268	34	64	NM_026189.3	Eepd1	mRNA	chr9:25481524-25489939	+	mmu:67484	Endonuclease/exonuclease/phosphatase family domain-containing protein 1
mmu-miR-10a-5p	Kcnj5	Miranda_RNAhybrid	-22.38	153	539	560	-27.9	0.530774	1889	1913	NM_010605.4	Kcnj5	mRNA	chr9:32314706-32344380	-	mmu:16521	Potassium inwardly-rectifying channel subfamily J member 5
mmu-miR-10a-5p	Tmprss5	Miranda_RNAhybrid	-27.34	161	478	500	-30	0.036427	482	501	NM_030709.2	Tmprss5	mRNA	chr9:49080719-49117591	+	mmu:80893	Transmembrane protease, serine 5 (Spinesin)

mmu-miR-10a-5p	Prtg	Miranda_RNAhybrid	-21.67	151	1785	1807	-26.3	0.923548	1781	1808	NM_175485.4	Prtg	mRNA	chr9:72807274-72917307	+	mmu:235472	Protogenin
mmu-miR-10a-5p	Tfdp2	Miranda_RNAhybrid	-22.26	155	4878	4900	-25.2	0.988859	4877	4901	NM_178667.4	Tfdp2	mRNA	chr9:96196246-96323646	+	mmu:211586	Transcription factor Dp-2
mmu-miR-10a-5p	Twf2	Miranda_RNAhybrid	-27.24	157	23	44	-27.2	0.076524	25	44	NM_011876.3	Twf2	mRNA	chr9:106203108-106215387	+	mmu:23999	Twinfilin-2
mmu-miR-10a-5p	Tmppe	Miranda_RNAhybrid	-20.79	151	3579	3599	-28.4	0.679376	243	271	NM_001200002.1	Tmppe	mRNA	chr9:114401095-114411201	+	mmu:100504715	Protein Tmppe
mmu-miR-10a-5p	Rbms3	Miranda_RNAhybrid	-21.86	163	3789	3812	-26.5	0.937595	3791	3812	NM_178660.4	Rbms3	mRNA	chr9:116572750-118040355	-	mmu:207181	Rbms3 protein
mmu-miR-10a-5p	Ctdspl	Miranda_RNAhybrid	-21.91	153	94	116	-27.2	0.710993	2657	2689	NM_133710.3	Ctdspl	mRNA	chr9:118929872-119044119	+	mmu:69274	CTD small phosphatase-like protein
mmu-miR	Atl3	Miranda_R	-23.35	154	1794	1816	-25.3	0.9718	1795	1816	NM_1460	Atl3	mRN	chr19:7494040-7	+	mmu:109	Atlastin-3

-10a -5p		NAhy brid									91.4		A	538609		168	
mmu -miR -10a -5p	Syt7	Miran da_R NAhy brid	-25. 78	158	2246	2269	-27.5	0.775 259	2246	2270	NM_ 1730 68.2	Syt7	m RN A	chr19:10 389090- 1045318 1	+	mmu :545 25	Synaptotag min-7
mmu -miR -10a -5p	Prpf 19	Miran da_R NAhy brid	-21. 65	150	2073	2093	-26.8	0.845 199	2070	2094	NM_ 1341 29.4	Prpf 19	m RN A	chr19:10 898645- 1090955 9	+	mmu :280 00	Pre-mRNA-processing factor 19
mmu -miR -10a -5p	Abli m1	Miran da_R NAhy brid	-32. 3	179	3682	3707	-34	0.079 7	3682	3707	NM_ 1786 88.3	Abli m1	m RN A	chr19:57 033264- 5721603 2	-	mmu :226 251	Ablim1 protein
mmu -miR -10a -5p	Mya dm	Miran da_R NAhy brid	-23. 77	158	1365	1386	-27.9	0.334 279	1361	1387	NM_ 0169 69.2	Mya dm	m RN A	chr7:329 2953-32 99355	+	mmu :509 18	Myeloid-ass ociated differentiati on marker
mmu -miR -10a -5p	Arhg ap35	Miran da_R NAhy brid	-26. 33	164	2238	2261	-26.7	0.754 374	2239	2262	NM_ 1727 39.4	Arhg ap35	m RN A	chr7:164 94473-1 6614993	-	mmu :232 906	Rho GTPase-acti vating protein 35
mmu -miR -10a -5p	Smg 9	Miran da_R NAhy brid	-24. 67	156	166	192	-26	0.141 912	167	193	NM_ 0280 47.2	Smg 9	m RN A	chr7:243 99926-2 4422777	+	mmu :719 97	Protein SMG9

mmu-miR-10a-5p	Zfp27	Miranda_RNAhybrid	-22.09	166	564	586	-26.4	0.260467	553	587	NM_011754.2	Zfp27	mRNA	chr7:29893337-29906532	-	mmu:22689	Mszf76
mmu-miR-10a-5p	Tyrobp	Miranda_RNAhybrid	-20.2	172	91	113	-27.1	0.020515	90	114	NM_011662.2	Tyrobp	mRNA	chr7:30413788-30417582	+	mmu:22177	TYRO protein tyrosine kinase-binding protein
mmu-miR-10a-5p	Rhpn2	Miranda_RNAhybrid	-21.61	166	844	865	-28.9	0.167764	835	866	NM_027897.4	Rhpn2	mRNA	chr7:35334237-35392287	+	mmu:52428	Rhpn2 protein
mmu-miR-10a-5p	Chrna7	Miranda_RNAhybrid	-25.07	156	234	260	-26.8	0.14323	236	261	NM_007390.3	Chrna7	mRNA	chr7:63098692-63212526	-	mmu:11441	Neuronal acetylcholine receptor subunit alpha-7
mmu-miR-10a-5p	Chd2	Miranda_RNAhybrid	-20.12	154	418	439	-29.8	0.298041	1472	1495	NM_001081345.2	Chd2	mRNA	chr7:73426652-73541746	-	mmu:244059	Chromodomain-helicase-DNA-binding protein 2
mmu-miR-10a	Crtc3	Miranda_RNAhy	-20.99	153	2251	2273	-25.2	0.926077	1699	1721	NM_173863.2	Crtc3	mRNA	chr7:80586627-80688942	-	mmu:70461	CREB-regulated transcription

-5p		brid															coactivator 3
mmu-miR-10a-5p	Olfr658	Miranda_RNAhybrid	-21.88	157	1135	1157	-25.3	0.696116	1132	1158	NM_147049.4	Olfr658	mRNA	chr7:104642880-104647305	-	mmu:259051	Olfactory receptor
mmu-miR-10a-5p	Iqck	Miranda_RNAhybrid	-21.23	153	454	474	-26.2	0.824508	453	474	NM_001081446.1	Iqck	mRNA	chr7:118855753-118915574	+	mmu:434232	Protein Iqck
mmu-miR-10a-5p	Ano1	Miranda_RNAhybrid	-20.54	159	240	262	-27.2	0.377973	237	263	NM_178642.5	Ano1	mRNA	chr7:144588549-144752045	-	mmu:101772	Anoctamin-1
mmu-miR-10a-5p	Rpl31	Miranda_RNAhybrid	-30.23	177	75	97	-33.2	0.007008	80	98	NM_053257.3	Rpl31	mRNA	chr1:39367851-39371911	+	mmu:114641	MCG126194, isoform CRA_b
mmu-miR-10a-5p	Mars2	Miranda_RNAhybrid	-27.93	168	980	1003	-27.2	0.275374	981	1004	NM_175439.3	Mars2	mRNA	chr1:55237177-55240058	+	mmu:212679	Methionine-tRNA synthetase 2 (Mitochondrial)
mmu-miR-10a	Aox2	Miranda_RNAhy	-21.37	153	299	321	-26.9	0.190926	291	322	NM_00100841	Aox2	mRNA	chr1:58278326-58379264	+	mmu:213043	Aldehyde oxidase 2

-5p		brid									9.2						
mmu-miR-10a-5p	Tns1	Miranda_RNAhybrid	-21.47	153	669	689	-25.3	0.965733	664	690	NM_177169.2	Tns1	mRNA	chr1:73910232-74098332	-	mmu:21961	Protein Tns1
mmu-miR-10a-5p	Cnot9	Miranda_RNAhybrid	-21.73	156	1111	1133	-25.1	0.823993	1099	1133							
mmu-miR-10a-5p	Hdac4	Miranda_RNAhybrid	-25.32	168	2303	2329	-29.1	0.525638	2305	2330	NM_207225.1	Hdac4	mRNA	chr1:91928779-92180231	-	mmu:208727	Histone deacetylase 4
mmu-miR-10a-5p	Tnfrsf11a	Miranda_RNAhybrid	-22.07	168	1560	1583	-26	0.843111	1557	1583	NM_009399.3	Tnfrsf11a	mRNA	chr1:105780747-105845798	+	mmu:21934	Tumor necrosis factor receptor superfamily member 11A
mmu-miR-10a-5p	Ptpn4	Miranda_RNAhybrid	-21.08	158	6627	6649	-25.4	0.99437	6624	6650	NM_019933.2	Ptpn4	mRNA	chr1:119652467-119837457	-	mmu:19258	Tyrosine-protein phosphatase non-recept

																	or type 4
mmu-miR-10a-5p	Pou2f1	Miranda_RNAhybrid	-21.11	153	25	47	-26.4	0.991216	22	48	NM_198934.3	Pou2f1	mRNA	chr1:165865150-165953071	-	mmu:18986	POU domain, class 2, transcription factor 1
mmu-miR-10a-5p	Hnrnpu	Miranda_RNAhybrid	-20.6	161	2916	2937	-26.7	0.961819	7717	7741	NM_016805.2	Hnrnpu	mRNA	chr1:178321108-178337810	-	mmu:51810	Protein Gm28062
mmu-miR-10a-5p	Grm3	Miranda_RNAhybrid	-22.2	157	262	285	-27.3	0.11312	258	285	NM_181850.2	Grm3	mRNA	chr5:9485236-9725352	-	mmu:108069	Grm3 protein
mmu-miR-10a-5p	Htr5a	Miranda_RNAhybrid	-22.92	166	1447	1469	-26.9	0.80253	1443	1469	NM_008314.2	Htr5a	mRNA	chr5:27841702-27853143	+	mmu:15563	5-hydroxytryptamine receptor 5A
mmu-miR-10a-5p	Nat8l	Miranda_RNAhybrid	-20.78	160	143	166	-25.6	0.962235	143	166	NM_001001985.3	Nat8l	mRNA	chr5:33995984-34005916	+	mmu:269642	N-acetylaspartate synthetase
mmu-miR	Golga3	Miranda_R	-21.56	155	1187	1214	-27	0.732723	1187	1214	NM_0081	Golga3	mRN	chr5:110176658-	+	mmu:269	Golgin subfamily A

-10a-5p		NAhybrid									46.3		A	110226470		682	member 3
mmu-miR-10a-5p	Ddx51	Miranda_RNAhybrid	-21.63	168	1988	2013	-25.7	0.858299	1989	2014	NM_027156.3	Ddx51	mRNA	chr5:110653451-110660496	+	mmu:69663	ATP-dependent RNA helicase DDX51
mmu-miR-10a-5p	Slc8b1	Miranda_RNAhybrid	-20.86	161	336	358	-25.7	0.443352	324	359	NM_133221.2	Slc8b1	mRNA	chr5:120511168-120527814	+	mmu:170756	Sodium/potassium/calcium exchanger 6, mitochondrial
mmu-miR-10a-5p	Pvrig	Miranda_RNAhybrid	-22.8	163	1457	1482	-27.2	0.815313	1462	1483	Pvrig-ps	Pvrig-ps	mRNA	chr5:138341770-138359800	+		poliovirus receptor related immunoglobulin domain containing, pseudogene
mmu-miR-10a-5p	Katnal1	Miranda_RNAhybrid	-25.36	168	863	886	-26.9	0.8411	852	887	NM_153572.2	Katnal1	mRNA	chr5:148871584-148928737	-	mmu:231912	Katanin p60 ATPase-containing subunit A-like 1

mmu-miR-10a-5p	Myt1l	Miranda_RNAhybrid	-23.34	165	1617	1639	-27.3	0.604913	1617	1639	NM_008666.3	Myt1l	mRNA	chr12:29533358-29923216	+	mmu:17933	Myelin transcription factor 1-like protein
mmu-miR-10a-5p	Ift43	Miranda_RNAhybrid	-23.28	151	1000	1022	-26.9	0.792936	1001	1023	NM_029601.3	Ift43	mRNA	chr12:86082561-86162459	+	mmu:76411	Intraflagellar transport protein 43 homolog
mmu-miR-10a-5p	Gpatch2l	Miranda_RNAhybrid	-22.07	154	2079	2103	-25.9	0.966385	2078	2103	NM_027405.2	Gpatch2l	mRNA	chr12:86241838-86291793	+	mmu:70373	Putative uncharacterized protein
mmu-miR-10a-5p	Bdkrb1	Miranda_RNAhybrid	-24.13	152	177	198	-30.8	0.005117	174	199	NM_007539.2	Bdkrb1	mRNA	chr12:105604091-105605428	+	mmu:12061	MCG1159
mmu-miR-10a-5p	Rcor1	Miranda_RNAhybrid	-21.66	151	2002	2024	-27.4	0.730884	1998	2025	NM_198023.2	Rcor1	mRNA	chr12:111039798-111113389	+	mmu:217864	REST corepressor 1
mmu-miR-10a-5p	Zfp386	Miranda_RNAhybrid	-23.99	155	2330	2352	-26.2	0.768888	2329	2353	NM_019565.4	Zfp386	mRNA	chr12:116047724-116063209	+	mmu:56220	Zinc finger protein 386 (Kruppel-like) isoform a
mmu-miR	Hic2	Miranda_R	-22.44	156	322	347	-25.6	0.948006	322	347	NM_1789	Hic2	mRN	chr16:17233587-	+	mmu:581	Hypermethylated in

-10a -5p		NAhy brid									22.3		A	1726343 0		80	cancer 2 protein
mmu -miR -10a -5p	Crkl	Miran da_R NAhy brid	-22. 69	160	2841	2864	-26.6	0.813 342	2839	2865	NM_ 0077 64.5	Crkl	m RN A	chr16:17 451985- 1748744 0	+	mmu :129 29	V-crk sarcoma virus CT10 oncogene homolog (Avian)-like
mmu -miR -10a -5p	Senp 5	Miran da_R NAhy brid	-22. 45	150	2005	2026	-25.3	0.949 225	2005	2026	NM_ 1771 03.4	Senp 5	m RN A	chr16:31 961906- 3201074 4	-	mmu :320 213	Sentrin-spe cific protease 5
mmu -miR -10a -5p	Hsp bap 1	Miran da_R NAhy brid	-21. 53	168	881	903	-25.8	0.862 532	883	903	NM_ 1751 11.3	Hsp bap 1	mi sc RN A	chr16:35 769655- 3582650 4	+	mmu :666 67	HSPB1-asso ciated protein 1
mmu -miR -10a -5p	Gtf2 e1	Miran da_R NAhy brid	-23. 09	155	1200	1224	-25.8	0.632 564	1202	1225	NM_ 0288 12.3	Gtf2 e1	m RN A	chr16:37 509796- 3753976 9	-	mmu :741 97	Putative uncharacteri zed protein
mmu -miR -10a -5p	Itsn1	Miran da_R NAhy brid	-25. 79	155	607	630	-28.7	0.802 109	605	631	NM_ 0105 87.2	Itsn1	m RN A	chr16:91 803274- 9192059 1	+	mmu :164 43	MAA-13 protein
mmu -miR	Atp8 a2	Miran da_R	-21. 28	168	1486	1510	-25.4	0.736 295	1487	1511	NM_ 0158	Atp8 a2	m RN	chr14:59 647741-	-	mmu :507	Phospholipi d-transporti

-10a-5p		NAhybrid									03.2		A	60086834		69	ng ATPase IB
mmu-miR-10a-5p	Jmjd8	Miranda_RNAhybrid	-23.42	159	384	405	-26.4	0.438114	381	405	NM_028101.4	Jmjd8	mRNA	chr17:25829043-25831843	+	mmu:72106	JmjC domain-containing protein 8
mmu-miR-10a-5p	D17Wsu92e	Miranda_RNAhybrid	-23.87	159	1234	1259	-26.1	0.788438	1236	1259	NM_001271511.1	D17Wsu92e	mRNA	chr17:27751232-27820542	-	mmu:224647	Uncharacterized protein C6orf106 homolog
mmu-miR-10a-5p	Zfp871	Miranda_RNAhybrid	-23.21	154	8698	8720	-25.9	0.992993	8702	8720	NM_172458.3	Zfp871	mRNA	chr17:32765496-32788287	-	mmu:208292	Protein Zfp871
mmu-miR-10a-5p	Zfp799	Miranda_RNAhybrid	-23.43	155	2478	2506	-26.7	0.820563	2478	2507	NM_177359.5	Zfp799	mRNA	chr17:32815449-32830261	-	mmu:240064	Protein Zfp799
mmu-miR-10a-5p	Zfp870	Miranda_RNAhybrid	-24.45	150	2085	2105	-27.5	0.662876	2085	2105	NM_207245.2	Zfp870	mRNA	chr17:32879221-32891433	-	mmu:240066	Protein Zfp870
mmu-miR-10a-5p	C4b	Miranda_RNAhybrid	-23.72	168	55	77	-27	0.008255	46	78	NM_009780.2	C4b	mRNA	chr17:34728380-34743897	-	mmu:12268	Complement component 4B (Childo

																	blood group)
mmu-miR-10a-5p	C4a	Miranda_RNAhybrid	-22.5	164	55	77	-26.5	0.010902	48	78	NM_011413.2	C4a	mRNA	chr17:34809092-34823454	-	mmu:625018	C4a protein
mmu-miR-10a-5p	Nfya	Miranda_RNAhybrid	-20.23	152	2215	2237	-25.8	0.788622	1247	1275	NM_010913.2	Nfya	mRNA	chr17:48386885-48409901	-	mmu:18044	Nuclear transcription factor Y subunit alpha
mmu-miR-10a-5p	Lbh	Miranda_RNAhybrid	-30.14	156	1249	1274	-32.5	0.084434	1248	1275	NM_029999.4	Lbh	mRNA	chr17:72918305-72941946	+	mmu:77889	Protein LBH
mmu-miR-10a-5p	Kcng3	Miranda_RNAhybrid	-22.51	167	3790	3812	-26	0.986205	3791	3812	NM_153512.1	Kcng3	mRNA	chr17:83579949-83631993	-	mmu:225030	Potassium voltage-gated channel subfamily G member 3
mmu-miR-10a-5p	Msrb2	Miranda_RNAhybrid	-24.74	155	191	219	-25.9	0.260059	192	220	NM_029619.2	Msrb2	mRNA	chr2:19371411-19380814	+	mmu:76467	Methionine-R-sulfoxide reductase B2, mitochondrial

mmu-miR-10a-5p	Gad2	Miranda_RNAhybrid	-23.92	168	3150	3173	-27.1	0.732273	3150	3173	NM_008078.2	Gad2	mRNA	chr2:22621422-22656274	+	mmu:14417	Glutamate decarboxylase 2
mmu-miR-10a-5p	Zer1	Miranda_RNAhybrid	-25.18	151	58	84	-30.2	0.1185	52	85	NM_178694.4	Zer1	mRNA	chr2:30106732-30125258	-	mmu:227693	Zyg-11 homolog B (C. elegans)-like
mmu-miR-10a-5p	Smtnl1	Miranda_RNAhybrid	-21.92	156	792	817	-26.1	0.377746	790	818	NM_024230.2	Smtnl1	mRNA	chr2:84811176-84822652	-	mmu:68678	Smoothelin-like protein 1
mmu-miR-10a-5p	Mapkbp1	Miranda_RNAhybrid	-21.64	154	364	386	-25.2	0.823562	362	387	NM_011941.3	Mapkbp1	mRNA	chr2:119972390-120027403	+	mmu:26390	Mitogen-activated protein kinase-binding protein 1
mmu-miR-10a-5p	LOC108168734	Miranda_RNAhybrid	-24.9	163	371	393	-26.9	0.103756	373	394							
mmu-miR-10a-5p	Dbndd2	Miranda_RNAhybrid	-28.63	151	1734	1762	-30	0.274627	1733	1762	NM_026797.2	Dbndd2	mRNA	chr2:164467153-164493319	+	mmu:52840	Dysbindin domain-containing protein 2

mmu-miR-10a-5p	Zmat2	Miranda_RNAhybrid	-20.71	155	1134	1161	-27.5	0.380433	1133	1161	NM_025594.3	Zmat2	mRNA	chr18:36793923-36799660	+	mmu:66492	Zinc finger matrin-type protein 2
mmu-miR-10a-5p	Slc25a2	Miranda_RNAhybrid	-27.92	172	210	232	-32.3	0.003294	209	233	NM_001159275.1	Slc25a2	mRNA	chr18:37637378-37638723	-	mmu:83885	Mutant ornithine transporter 2
mmu-miR-10a-5p	Spry4	Miranda_RNAhybrid	-25.29	165	247	267	-27.6	0.657235	247	267	NM_011898.2	Spry4	mRNA	chr18:38586265-38601268	-	mmu:24066	Protein sprouty homolog 4
mmu-miR-10a-5p	Ticam2	Miranda_RNAhybrid	-20.92	160	1388	1412	-25.2	0.826137	1389	1412	NM_173394.3	Ticam2	mRNA	chr18:46558231-46574533	-	mmu:225471	TIR domain-containing adapter molecule 2
mmu-miR-10a-5p	Ppargc1b	Miranda_RNAhybrid	-23.75	154	4066	4088	-26.9	0.942964	4041	4089	NM_133249.2	Ppargc1b	mRNA	chr18:61291445-61401462	-	mmu:170826	Peroxisome proliferative activated receptor, gamma, coactivator 1 beta
mmu-miR	Rnf165	Miranda_R	-22.57	160	3689	3712	-25	0.994871	2893	2916	NM_0011	Rnf165	mRN	chr18:77456110-	-	mmu:225	RING finger protein 165

-10a-5p		NAhybrid									64504.1		A	77565136		743	
